# Phospholipase C: underrated players in microbial infections

**DOI:** 10.3389/fcimb.2023.1089374

**Published:** 2023-04-17

**Authors:** Vinayak Singh, Rupal Rai, Bijina J. Mathew, Rashmi Chourasia, Anirudh K. Singh, Awanish Kumar, Shivendra K. Chaurasiya

**Affiliations:** ^1^ Molecular Signalling Lab, Department of Biological Science and Engineering, Maulana Azad National Institute of Technology, Bhopal, Madhya Pradesh, India; ^2^ Department of Chemistry, IES University, Bhopal, Madhya Pradesh, India; ^3^ School of Sciences, SAM Global University, Raisen, Madhya Pradesh, India; ^4^ Department of Biotechnology, National Institute of Technology, Raipur, Chhattisgarh, India

**Keywords:** bacterial infections, cell signaling, phospholipase C, tuberculosis, phospholipids (PL), *Listeria monocytogenes*, *Helicobabacter pylori*, *Clostridium perfringens*

## Abstract

During bacterial infections, one or more virulence factors are required to support the survival, growth, and colonization of the pathogen within the host, leading to the symptomatic characteristic of the disease. The outcome of bacterial infections is determined by several factors from both host as well as pathogen origin. Proteins and enzymes involved in cellular signaling are important players in determining the outcome of host–pathogen interactions. phospholipase C (PLCs) participate in cellular signaling and regulation by virtue of their ability to hydrolyze membrane phospholipids into di-acyl-glycerol (DAG) and inositol triphosphate (IP3), which further causes the activation of other signaling pathways involved in various processes, including immune response. A total of 13 PLC isoforms are known so far, differing in their structure, regulation, and tissue-specific distribution. Different PLC isoforms have been implicated in various diseases, including cancer and infectious diseases; however, their roles in infectious diseases are not clearly understood. Many studies have suggested the prominent roles of both host and pathogen-derived PLCs during infections. PLCs have also been shown to contribute towards disease pathogenesis and the onset of disease symptoms. In this review, we have discussed the contribution of PLCs as a determinant of the outcome of host-pathogen interaction and pathogenesis during bacterial infections of human importance.

## Introduction

In all living organisms, phospholipids are present as a major component of all cellular membranes, along with cholesterol and glycolipids. The phospholipid membrane separates the extracellular and intracellular environments and serves as a scaffold for the membrane-associated proteins. Membrane phospholipids and their hydrolytic products play a significant role in cellular signaling (Pelech and Vance, 1989; Exton, 1990; Kumari et al., 2018). Phospholipids have structural variation in the hydrophilic head group [such as phosphatidylinositol (PI) or phosphatidylcholine (PC)] and are attached to fatty acid chains of varying length and saturation. Variability in the structure of phospholipids enables them to play different functions in the cell membrane and cell signaling (Verkleij et al., 1973).

**Figure 1:**
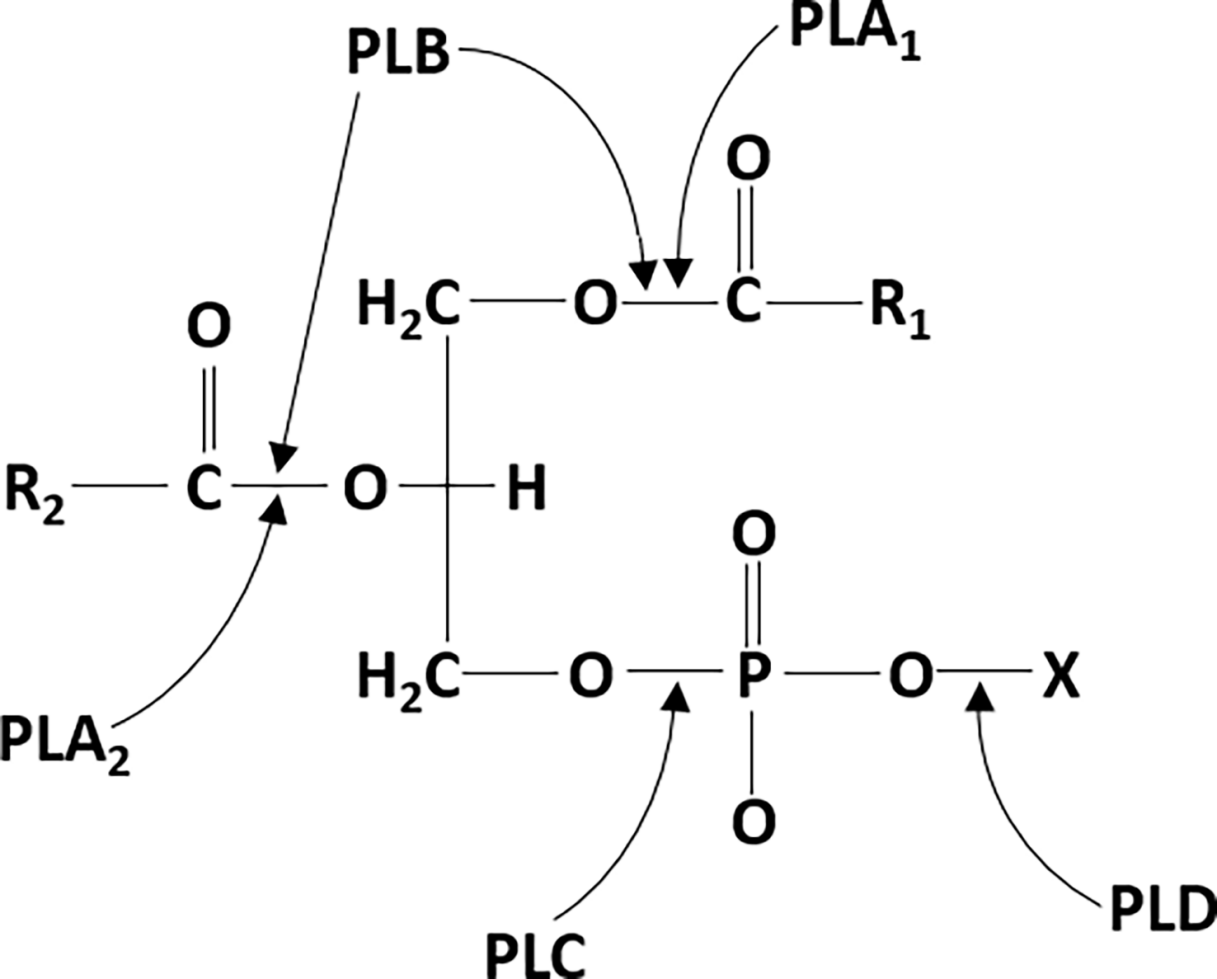
phospholipase cleavage sites on phospholipid substrates: phospholipase A1 (PLA1) and phospholipase A2 (PLA2) cleave the ester bond at the sn-1 and sn-2 positions of phospholipids, respectively. Phospholipase B (PLB) can also hydrolyze at both the sn-1 and sn-2 positions. phospholipase C (PLC) hydrolyzes the phosphodiester bond between the glycerol backbone and the phosphate group. Phospholipase D (PLD) hydrolyzes the phosphodiester bond on the polar side to remove the head group.

Phospholipases (PLs) are the ubiquitous group of enzymes that catalyze the hydrolysis of phospholipids by cleaving the ester bond. After hydrolysis, secondary messengers are generated, which are further involved in cell signaling, membrane trafficking, cell proliferation, etc (Barrett-Bee et al., 1985; Cox et al., 2001; Sato et al., 2016). PLs have different sites of cleavage on phospholipid molecules, therefore the PLs are classified as PLA, PLB, PLC, and PLD (**Figure 1**). The PLA are acyl hydrolases and are further classified into two subtypes: PLA1 and PLA2. PLA1 cleaves the acyl ester bond at the sn-1 position to produce free fatty acids and lysophospholipids, whereas PLA2 cleaves at the sn-2 position to produce arachidonic acid and lysophosphatidic acid (Scandella and Kornberg, 1971; Vasquez et al., 2018). PLB can hydrolyze both the acyl groups, and its lysophospholipase activity is responsible for cleaving the bond between the fatty acid and the lysophospholipid (Leidich et al., 1998; Wilton, 2008). PLCs cleave the phosphodiester bond between the glycerol backbone and the phosphate group to produce diacylglycerol (DAG) and a phosphate-containing head group (Suh et al., 2008). PLD catalyzes the removal of the phospholipid head group on the polar side to produce phosphatidic acid and alcohol. Therefore, PLC and PLD are phosphodiesterases (Suh et al., 2008; Abdulnour et al., 2018). In this review, we mainly focus on the role of PLCs in bacterial infections.

On the basis of the type of organisms, the PLCs are categorized as bacterial PLCs and mammalian PLCs. In 1941, Macfarlane and Knight were the first to demonstrate that the α-toxin of *Clostridium perfringens* was a PLC. Since then, PLCs have been recognized as potential virulence factors required during the pathogenesis of several bacteria (Titball, 1993; Songer, 1997; Kumari et al., 2018). While PLCs are produced by several pathogenic and nonpathogenic bacteria, the focus of this review is on the PLCs of some of the most important human pathogens (Titball, 1993; Songer, 1997). PLCs can be further grouped on the basis of the preferred substrates they utilize (Titball, 1993). Accordingly, PLCs can be PI-PLCs, PC-PLCs, and nonspecific PLCs (Aloulou et al., 2018). PI-PLCs have been detected in a wide variety of organisms, including bacteria, plants, and animals. Prokaryotic PI-PLCs consist of a single domain of 30–35 kDa and act as a virulence factor in several pathogenic bacteria. Eukaryotic PI-PLCs are large (80–150 kDa) and consist of several distinct domains that play predominant roles in signal transduction and generate inositol 1,4,5-triphosphate (IP3) and DAG by the hydrolysis of phosphatidylinositol 4,5-bisphosphate (PIP2). Thus, PLCs regulate the concentrations of PIP2, IP3, and DAG within the cell, which play critical roles in cellular signaling (Heinz et al., 1998; Kadamur and Ross, 2013). For optimal catalytic activity, bacterial and mammalian PLCs require calcium ions (Ca^2+^) and zinc ions (Zn^2+^), respectively, as cofactors (Heinz et al., 1998; Aurass et al., 2013).

## Mechanism of PLC activation

**Figure 2:**
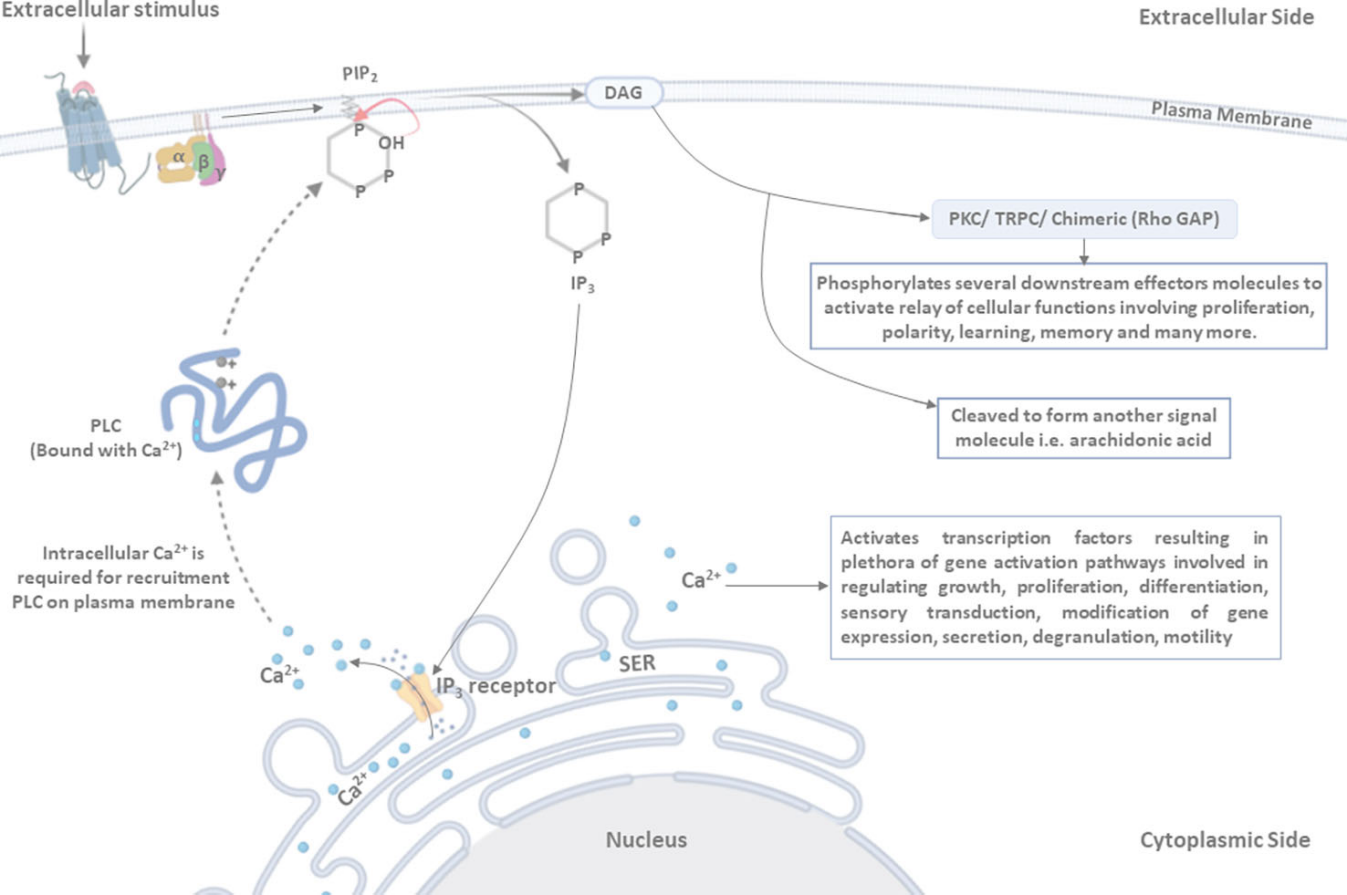
Mechanism of PLC activation: ligand binding induces conformational changes in the receptor, leading to the activation of associated PLC. Activated PLC hydrolyzes the membrane-associated phosphatidylinositol 4,5-bisphosphate (PIP2) into di-acyl-glycerol (DAG) and inositol triphosphate (IP3). DAG, being a hydrophobic molecule, remains in the membrane and activates the protein kinase C (PKC)/transient receptor potential channel (TRPC)/chimeric (Rho GAP) proteins, resulting in the phosphorylation of several downstream effector molecules that are involved in regulating the relay of cellular functions (such as proliferation, polarity, learning, memory, and many more). DAG may also be cleaved to form another signal molecule, i.e., arachidonic acid. IP3 is released in the cytoplasm and binds to its receptor present on the smooth endoplasmic reticulum (SER) membrane (IP3 receptor), as a result of which Ca^2+^ is released in cytosol from the SER. PLC, in association with intracellular Ca^2+^, is recruited to the plasma membrane, where it interacts with and cleaves the PIP2 molecule. Two positively charged amino acid residues are present in the active site of PLC and play a critical role in interaction with the four and five positions of the inositol ring to hydrolyze the PIP2 molecule. Furthermore, the 2’-hydroxyl group attacks the 1’-phosphate in order to release IP3 in the cytosol.

PLC in association with Ca^2+^ is recruited to the plasma membrane, where it interacts with the hydrophilic domain of PIP2 (specifically with the inositol ring), which is flanked toward the cytosol (Fukami et al., 2010; Sekiya, 2013). The active site of PLC has positively charged conserved amino acid residues, which are critically required to interact with the four and five positions of the inositol ring in PIP2 (Kim et al., 1991; Fanning and Anderson, 1996; Gifford et al., 2007; Wang et al., 2010; Sekiya, 2013). The interaction of PLC with PIP2 activates the 2-hydroxy group of the inositol ring, which further attacks 1-phosphate, leading to the formation of 1,2-cyclic phosphate and DAG intermediates. These cyclic intermediates get converted into an acyclic inositol derivative, which is referred to as IP3 (Sekiya, 2013). DAG, being a hydrophobic molecule, resides within the plasma membrane (**Figure 2**). IP3 and DAG act as secondary messengers and participate in downstream signaling processes (Titball, 1998; Williams, 1999; Wilton, 2008; Kadamur and Ross, 2013; Sekiya, 2013; Stahelin, 2016).

## Structure of different PLC isoforms

**Figure 3:**
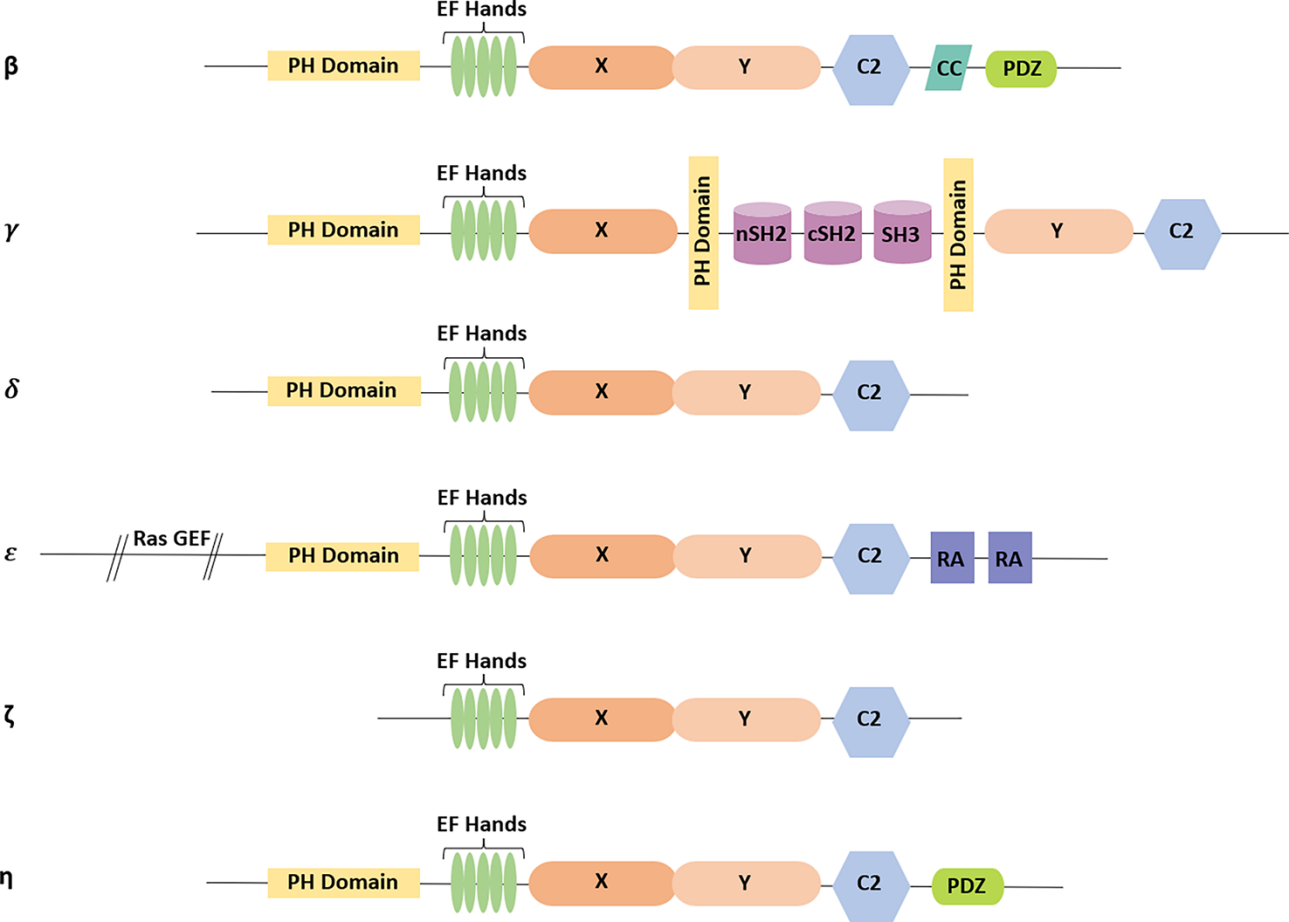
Domain architecture present in different isoforms of PLC: all PLC isoforms have the core domains consisting of pleckstrin homology (PH) domain, EF-hand motifs, X and Y domains, and C2 domain (except in PLC-ζ). Additionally, CC and PDZ domains are present in PLC-β isoform. In PLC-γ, X and Y domains are separated by two src homology 2 (SH2) domains followed by one src homology 3 (SH3) domain, which are present in between the additional PH domains. PLC-δ comprises only the core domains. The Ras guanine exchange factor (GEF) domain is at the N-terminal tail, and the two RA domains are present at the C-terminal tail in PLC-ε. PLC-ζ does not contain a PH domain. PLC-η consists of the core domains followed by the PDZ domain at the C-terminal tail.

In mammals, 13 different PLC isozymes have been reported and are divided into six different subfamilies, namely β, γ, δ, ε, ζ, and η (Suh et al., 2008; Bunney and Katan, 2011). The structural organization of PLC isozymes consists of various domains and motifs (Sekiya, 2013). However, the pleckstrin homology (PH) domain, EF-hand motifs, X and Y domains, and C2 domains show more than 40%–50% similarity (Fukami et al., 2010). Core domains are present in all isoforms, whereas PLC-ζ is the only exception that does not contain the PH domain (**Figure 3**) (Bunney and Katan, 2011).

### PH domains

The N-termini of PLCs (except PLC-ζ) have the PH domain, which consists of ~120 amino acids. Several other protein families involved in signal transduction commonly have the PH domain in their structure (Harlan et al., 1994). The PH domain of PLC, in association with Ca^2+^, is recruited to the plasma membrane to establish the interaction with phosphoinositides (Sekiya, 2013).

### EF-hand motifs

The catalytic core of PLCs is composed of the EF-hand motifs, X and Y domains, and C2 domain (Rhee and Choi, 1992). Crystallographic analysis of PLC-δ1 reveals that EF-hand motifs are helix-loop-helix motifs, generally present in calcium-binding proteins like calreticulin, calmodulin, and troponin (Essen et al., 1996). The binding of Ca^2+^ leads to a conformational change in the EF-hand motif, as a result of which PLC stabilizes and then exposes the binding site for other proteins to further initiate the calcium-regulated functions and helps in signal transduction (Rhee and Choi, 1992). The binding of Ca^2+^ promotes the interaction of the PH domain of PLC with PIP2 (Essen et al., 1997; Otterhag et al., 2001). However, it is not yet clear whether EF-hand motifs actually bind the Ca^2+^ (Sekiya, 2013).

### X and Y domains

Crystallographic studies of PLC-δ1, PLC-β2, and PLC-β3 revealed that the X and Y domains are composed of ~300 amino acids, which lie at the C-terminal of EF-hand motifs (Essen et al., 1996; Jezyk et al., 2006; Waldo et al., 2010). An alternative arrangement of α-β structures in the X and Y domain forms a βαβαβαβα motif with a triose phosphate isomerase (TIM) barrel-like structure (Essen et al., 1996). The X-region contains histidine (His^311^ and His^356^) as catalytic residues, which are required for the generation of 1,2-cyclic inositol 4,5-bisphosphate (Ellis MV and Katan, 1995; Essen et al., 1996; Bunney and Katan, 2011). These His^311^ and His^356^ residues are highly conserved across the PLC family members (Ellis MV and Katan, 1995). Structurally, the Y-region belongs to residues from 489 to 606 and plays an important part in substrate recognition (Ryu et al., 1987; Williams, 1999; Nagano et al., 2002). The X domain represents the first half while the Y domain represents the second half, of the TIM barrel-like structure (Ellis MV and Katan, 1995; Nagano et al., 2002). In PLC-γ, the X and Y regions are separated by the two src homology 2 (SH2) domains at the N-terminal, followed by the src homology 3 (SH3) domain. The SH2 domain of PLCγ serves as a docking site for phosphorylation and is activated by the tyrosine kinase growth factor receptor (Wahl et al., 1988; Meisenhelder et al., 1989; Margolis et al., 1990). The SH3 domain is responsible for the cellular localization of signaling proteins (Bar-Sagi et al., 1993; Gout et al., 1993).

### C2 domains

More than 40 different proteins involved in signal transduction and membrane interactions are reported to have the C2 domain. It consists of ~120 amino acids and has binding sites for several proteins, through which they are engaged in signal transduction and membrane interaction processes. These domains form an eight-stranded antiparallel β sandwich complex (Essen et al., 1996). Three to four C2 domains were reported in PLC-*δ* family members. The C2 domain operates in combination with Ca^2+^-mediated binding of PLC to anionic phospholipids and conducts signal transduction as well as membrane trafficking. Multiple binding sites for Ca^2+^ on PLCs act synergistically to mediate diverse functions (Essen et al., 1997).

### PDZ domains (postsynaptic density-95, *Drosophila* disc large tumor suppressor, and Zonula occludens-1 protein)

The PDZ domain is present at the C-terminal tail of PLC-β and PLC-η (Fanning and Anderson, 1996; Vines, 2012; Sekiya, 2013). The PDZ binding motifs are formed by two α-helices and five or six β-strands. PDZ domains are found in several signaling proteins other than PLCs (Fanning and Anderson, 1996). The PDZ domain serves as the binding site for large molecular complexes (Wang et al., 2010). Crystallographic and biochemical studies revealed that PDZ domains have two distinct mechanisms through which they interact either with the protein having the PDZ domain or bind with the C-terminal of an unrelated protein (Fanning and Anderson, 1996).

## Isoforms of PLC

### PLC-β (1/2/3/4)

Several physical and chemical stimuli are received by the cells *via* receptors that are coupled to heterotrimeric G-protein (G_α_, G_β_, and G_γ_). These stimuli lead to the activation of G_α_, causing PLC-β activation and the hydrolysis of PIP2 (Sekiya, 2013). There are four different isoforms of PLC-*β*, and the size varies from 130 to 152 kDa (Vines, 2012). PLC-β isoforms are stimulated by G-proteins and Ca^2+^. PLC-*β* is expressed in a tissue-specific manner and shows different sensitivities toward G-protein-mediated activation (Kadamur and Ross, 2013). Each β isoform has many splice variants, but the functions of many of these variants are unknown (Lagercrantz et al., 1995; Mao et al., 2000; Vines, 2012). The C-terminal of PLC-*β* isoforms shows some differences, and the PLC-β subfamily has a unique 450-amino acid long C-terminal extension, but the significance of this difference is unknown. This extension contains signals for nuclear and cytoplasmic localization of PLC-*β*1b and PLC-*β*1a, respectively (Vines, 2012). Regulation of PLC-*β* isoforms involves regulation by phosphatidic acid, a small GTP-binding protein (Rac), and site-specific phosphorylation by several kinases (Kadamur and Ross, 2013). Furthermore, PLC-*β* isoforms are involved in some physiological conditions such as epilepsy, abnormal functioning of neutrophils and platelets, hyper-responsiveness to opioid stimulation, ataxia, and impairment in visual perception (Vines, 2012).

### PLC-γ (1/2)

Two isoforms of PLC-γ are known as PLC-γ1 and PLC-γ2, with an approximate molecular weight of 140 kDa (Vines, 2012; Sekiya, 2013). Both isoforms are characterized by a large, unique multidomain X-Y linker that plays a significant role in its regulation and a split PH-domain including two SH2 domains (nSH2 and cSH2) and one SH3 domain. The structure and regulation of PLC-γ1 and PLC-γ2 seem to be similar in most cases (Kadamur and Ross, 2013). PLC-γ1 is expressed ubiquitously and appears to be important for controlling cell growth and differentiation. PLC-γ2 is primarily expressed in the cells of the hematopoietic lineage (Bonvini et al., 2003). Disruption of the PLC-γ1 gene in mice is fatal at an early embryonic stage. Loss of the PLC-γ2 gene is not fatal but results in impairment of B-cell maturation, leading to immunodeficient conditions and defective Fc-receptor-mediated signaling in platelets and mast cells (Sekiya, 2013).

### PLC-δ (1/3/4)

PLC-δ was originally identified in bovine as PLC-δ2, but later it was found to be homologous to humans and mouse PLC-δ4 (Irino et al., 2004; Sekiya, 2013). Three isoforms of PLC-δ (PLC-δ1, PLC-δ3, and PLC-δ4) have been identified with an approximate molecular weight of 90 kDa. PLC-δ isoforms have simpler structures compared to the other PLC isozymes (Meldrum et al., 1991; Irino et al., 2004; Sekiya, 2013). PLC-δ isoforms show greater sensitivity to Ca^2+^ in comparison to β and γ isozymes, suggesting Ca^2+^ is essential for δ-isozyme activity (Sekiya, 2013). PLC-δ is activated following an increase in the cytoplasmic Ca^2+^ concentration. The PH domain of PLC-δ binds PIP2 on the plasma membrane (Allen et al., 1997; Kim et al., 1999; Sekiya, 2013). After substrate hydrolysis, PLC-δ also interacts with IP3 with a higher affinity, causing the dissociation of membrane-bound PLC-δ and making it inactive, thus acting as a negative feedback regulation system (Sekiya, 2013). Although PLC-δ is not essential, misregulation of certain isoforms leads to some abnormal conditions. Deregulation of PLC-δ1 has been associated with Alzheimer’s disease and hypertension (Shimohama et al., 1991; Yagisawa et al., 1991). PLC-δ1 deficiency in mice caused them to look like nude mice. Expression of the PLC-δ1 was reportedly higher in hair follicles, and homozygous gene deletion resulted in hair loss (Ichinohe et al., 2007; Nakamura et al., 2008). Deletion of PLC-δ3 or PLC-δ4 genes showed no phenotypic effects in mice, but sperms from PLC-δ4-deficient mice showed a defective acrosomal reaction, a process required for fertilization (Fukami et al., 2001; Fukami et al., 2003).

### PLC-ε

PLC-ε, first identified in *Caenorhabditis elegans*, is the largest PLC isoform cloned to date, with a molecular weight of 250 kDa. The N-terminal of PLC-ε contains CDC 25-like guanine exchange factor (GEF) domain followed by core elements and the C2 domain followed by two copies of Ras binding (RA) domains. Later, this PLC isoform was cloned from human cells, where it has been recognized as a Ras-activated isoform that works in association with the RA domain. The RA domain of PLC-ε directly interacts with Ras, Ras family GTPases, and Rho (Kelley et al., 2001; Song et al., 2001). Regulation of the activity of PLC-ε by Ras and Rho suggests its role in the proliferation and migration of cells (Vines, 2012). Unlike other isoforms of PLC, it is activated not only by heterotrimeric G-protein but also by small GTPases (Song et al., 2001; Vines, 2012). Knockout of PLC-ε in mice showed impaired development of the heart and its functioning (Sekiya, 2013).

### PLC-ζ

PLC-ζ was first reported in the sperm head, where it serves a crucial role in the activation of oocytes during fertilization (Jones et al., 2000; Cox et al., 2002; Saunders et al., 2002; Fujimoto et al., 2004). Sperm-specific PLC-ζ regulates the oscillation of Ca^+2^ in the cytoplasm of fertilizing eggs (Cox et al., 2002; Saunders et al., 2002; Swann et al., 2006). PLC-ζ is the only PLC isoform without the PH domain and shows the closest homology with PLC-δ1 (Swann et al., 2006). The PH domain is not required for the membrane localization of PLC-ζ. However, it is not yet known how PLC-ζ associates with the cell membrane without the PH domain and catalyzes the reaction (Vines, 2012). Loss of PLC-ζ affects male fertility (Yoon et al., 2008; Heytens et al., 2009).

### PLC-η (1/2)

Two isoforms of PLC-*η*, i.e., PLC-η1 and PLC-η2 have been reported with an apparent molecular weight of 115 kDa and 125 kDa respectively (Hwang et al., 2005; Stewart et al., 2005; Zhou et al., 2005). Both show ~ 50% sequence homology with each other and are expressed in neurons and the brain (Hwang et al., 2005; Nakahara et al., 2005). PLC-η shows extremely higher sensitivity towards changes in the Ca^2+^ level, even when compared with PLC-*δ* (Zhou et al., 2005). In humans, deletion of chromosomal region encoding for PLC-η2 might be linked to intellectual disability (Lo Vasco, 2011).

## Role of PLC in bacteria

PLCs are essential virulence factors in many bacteria as they contribute to the avoidance of phagosomal maturation or phagosomal escape, tissue colonization, establishment of infection, and pathogenesis. In this section, we will discuss the specific roles of PLCs in various infections caused by pathogenic bacteria.

## Role of PLC in *Clostridium perfringens* infection

*Clostridium perfringens* (*C. perfringens*) is an anaerobic spore-forming bacterium extensively distributed in nature. *C. perfringens* is found in soil, sewage, and the gastrointestinal tract of humans and other animals (Flores-Díaz and Alape-Girón, 2003; Kiu and Hall, 2018). *C. perfringens* causes various human diseases such as gas gangrene or myonecrosis and represents the most severe, sudden onset gram-positive bacterial infection. Gas gangrene is a pathological condition when healthy, viable tissues are damaged rapidly. Amputation of the affected tissue is the only option to save the life of an infected individual (Monturiol-Gross et al., 2014).

*C. perfringens* PLC, also referred to as α-toxin, is a 42.5-kDa protein with 370-amino acid residues. Clostridial PLC is an extracellular Zn^2+^ metalloenzyme that mainly hydrolyzes PC and sphingomyelin (Naylor et al., 1998; Naylor et al., 1999; Alape-Girón et al., 2000). The α-toxin is a critically important virulence factor for the pathogenesis of gas gangrene. The α-toxin causes the aggregation of platelets and has myotoxic and cytotoxic lethal effects (Alape-Girón et al., 2000). 3-D structure analysis of α-toxin revealed that it is composed of two domains joined together by a short hinge region. The N-terminal domain (246 residues) comprises the active site consists of 10 tightly packed α-helices while the C-terminal domain (124 residues) is composed of four-stranded sandwiched β-sheets (Naylor et al., 1998; Naylor et al., 1999). The N-terminal domain is the catalytic domain for which Zn^2+^ is essential. The C-terminal domain shows strong structural similarities with the C2 domain of eukaryotic PLCs and is responsible for the binding of α-toxin to phospholipids on membranes in Ca^2+^-dependent manner. Such domains are frequently present in protein functioning as a secondary messenger (Naylor et al., 1998).

Some researchers suggest that α-toxin can be present in two conformations: the first one is an open conformation in which three Zn^2+^ ions are bound to make the active site accessible, while the second one is a closed conformation in which two Zn^2+^ ions are bound and the active site is masked by 135–150-amino acid residues (Alape-Girón et al., 2000; Flores-Díaz et al., 2005). Specific binding of the C-terminal domain to the target membrane leads to conformational changes within the structure of α-toxin. Through these changes, the active site is exposed, allowing catalysis to occur (Naylor et al., 1999).

*C. perfringens* enters inside the host *via* wounds and starts growing rapidly, producing α-toxin. α-toxin plays a key role in spreading the infection by suppressing the host’s immune response. α-toxin also triggers the release of inflammatory mediators and changes the intracellular Ca^2+^ level (Naylor et al., 1999). Calcium and zinc ions are essential for the α-toxin to bind with lipids on the cell membrane and hydrolyze the substrate, respectively (Moreau et al., 1988). The binding of calcium ions allows the formation of a hydrogen bond between Tyr62 of the N-terminal domain with Asn294 of the C-terminal domain, which is required for the C-terminal domain to communicate with the active site (Naylor et al., 1999).

Higher cellular levels of α-toxin disrupt the plasma membrane, causing cytolysis, while at low levels perturb phospholipid metabolism, impair various signal transduction processes, and induce the unregulated generation of secondary messengers (Flores-Díaz et al., 2004). Mutants with insertional inactivation of the α-toxin gene were not able to produce an active form of toxin and are unable to cause gas gangrene (Awad et al., 1995). Immunization of mice with a fragment of α-toxin consisting of the C-terminal domain protects them from a lethal myonecrosis infection (Naylor et al., 1999). These findings suggest that α-toxin of *C. perfringens* play a critical role in its pathogenesis (Awad et al., 1995).

α-toxin shows diverse roles depending on the cell types. In intestinal epithelial cells, it induces the release of arachidonic acid by activating the host-specific phospholipase A2; because of this event, PI gets degraded and the activation of protein kinase C (PKC) and calmodulin takes place. α-toxin elevates the levels of intracellular free Ca^2+^, which may be responsible for PKC activation (Gustafson and Tagesson, 1990). In rabbit neutrophils, α-toxin interacts with the tropomyosin receptor kinase A (TrkA receptor) and host PLC. While its interaction with TrkA phosphorylates the 3-phosphoinositide-dependent protein kinase 1 (PDK1), its interaction with the host PLC induces the formation of DAG. DAG and the PDK1 synergistically activate the PKC to produce the superoxide anions ( ${\text{O}}_{2}^{-}$ ) through the activation of the MEK/extracellular signal-regulated kinases (ERK) pathway (Oda et al., 2006).

α-toxin induces the recruitment of neutrophils on vascular endothelium, especially in the lungs and liver (Oda et al., 2012), leading to the formation of blood clots. This results in the reduced supply of oxygen in the tissues, favoring anaerobic conditions. This enhances the outspread of *C. perfringens* within the host tissues (Flores-Díaz et al., 2004).

α-toxin signal is communicated through membrane receptors and their nuclear or cytoplasmic targets *via* various pathways, including the MAPK pathway (Wortzel and Seger, 2011). The α-toxin simultaneously activates the ERK1/2-nuclear factor-kB (NF-kB) and p38 mitogen-activated protein kinases (MAPK) cascades, which promote the expression and stabilization of interleukin-8, respectively (Oda et al., 2012). Many cellular processes are regulated by the ERK1/2 signaling cascade, such as proliferation, differentiation, survival, and apoptosis, as well as the stress response (Wortzel and Seger, 2011). Deregulation of the ERK1/2 signaling cascade is associated with various diseases like neurodegenerative diseases, developmental diseases, diabetes, and cancer (Tanti and Jager, 2009; Tidyman and Rauen, 2009; Kim and Choi, 2010; Wortzel and Seger, 2011).

In diabetic animals, the UDP-glucose levels are low in tissues, and these tissues are hypersensitive to the cytotoxic effects of α-toxin through some unknown mechanism (Flores-Díaz et al., 1998). Gangliosides protect cell membrane disruption induced by α-toxin. Muscles generally have a lower amount of complex gangliosides among mammalian tissues, explaining their higher susceptibility to rapid and extensive damage caused by α-toxin and the release of creatine kinase in plasma (Flores-Díaz et al., 2005). The mechanism of ganglioside-mediated protection from α-toxin and the higher sensitivity of tissues with low UDP-glucose for α-toxin-mediated cytotoxicity remain to be explored.

### PLC regulates phagocytosis, intracellular survival, and immune response during mycobacterial infections

*Mycobacterium tuberculosis* (Mtb) is an acid-fast, rod-shaped, nonmotile, nonspore-forming, catalase-positive bacteria that ranges from 0.2 to 0.6μm in diameter and 1.0 to 10 μm in length. Different mycobacterial strains produce distinct morphological colonies varying from smooth to rough surfaces (Gordon and Parish, 2018). They also have distinct colony colors ranging from white to orange or pink (Iivanainen et al., 1999). Most of the mycobacterial strains are aerobic in nature, while some of them are microaerophilic (Falkinham, 1996).

Approximately 130 mycobacterial species are harmless to humans, but a few of them are among the major threats to human health and life. Mtb survives and multiplies inside the host macrophages by manipulating their machinery, leading to active tuberculosis (TB) development. Lipid metabolism is thought to be the central metabolic pathway among mycobacterial species (Bakala N'goma et al., 2010). Mycobacterial pathogens conceal themselves from the host’s defense system and harmonize their gene expression according to the intracellular environment (Sundararajan and Muniyan, 2021).

Mtb infects the macrophages by exploiting their phagocytic nature and is able to persist the infection in a state of dormancy by forming granulomas within the host (Wayne and Sohaskey, 2001). Before entering the dormant stage, Mtb starts to accumulate the lipids derived from the host cell membrane degradation and later hydrolyze them to reactivate the infection process. Lipolytic enzymes such as PLCs might be required for the degradation of the macrophage’s membrane (Côtes et al., 2008). Various studies on PLCs derived from both the host and the pathogen showed their significant role during bacterial pathogenesis (Songer, 1997; Raynaud et al., 2002; Poussin et al., 2009).

Mtb contains four closely related genes encoding putative PLCs; PLC-A, PLC-B, PLC-C, and PLC-D (Raynaud et al., 2002; Le Chevalier et al., 2015). PLC is a critical virulence factor and is important for persistent infection. Genetic studies using PLC mutant strains established that all four PLC genes encode functional enzymes that are capable of hydrolyzing their PC-like substrates (Raynaud et al., 2002).

*Mycobacterium smegmatis* (MS) was used as the expression system to produce active soluble recombinant PLC from Mtb that was expressed at very low levels, therefore the biochemical properties and the substrate specificity of Mtb’s PLCs are yet to be determined (Johansen et al., 1996). To analyze the role of mycobacterial PLC in macrophage cytotoxicity, Assis and co-workers used two different groups of isolates. Group A consisted of Mtb isolates expressing PLC, while group B consisted of Mtb isolates that do not express PLC; 24 h postinfection, groups A and B isolates of infected rat alveolar macrophages showed a 40% and 20% reduction in cell viability, respectively. This indicates that mycobacterial PLC has cytotoxic effects. Group A-infected host cells were detected with a higher number of kinase phosphorylated proteins, which are known to play several roles in intracellular signaling pathways. It was also observed that group A-infected cells released significantly higher levels of proinflammatory cytokines and nitric oxide (NO) as compared to group B-infected or noninfected cells. These data suggest that the alveolar macrophage activation and the production of pro-inflammatory cytokines might be induced by the PLC of mycobacterial origin. To evaluate the role of PLC in subverting host biosynthetic pathways as a strategy employed by mycobacteria to evade the host immune response, the mRNA expression of host enzymes and receptors involved (eicosanoid biosynthesis pathways like cyclooxygenase-2 (COX-2), prostaglandin E_2_ receptor 2 (EP-2) and prostaglandin E_2_ receptor 4 (EP-4), and leukotriene B4 (LTB4)) were analyzed. Expression of COX-2, EP-2, and EP-4 was found to be lower in group A-infected cells as compared to group B-infected cells, while higher expression of LTB4 was observed in group A-infected cells. These data suggest impairment of the eicosanoid biosynthesis pathway by mycobacterial PLC, which may help Mtb escape from the host’s immune response. No difference in apoptotic activity was observed in both groups A and B isolates, while necrosis was significantly higher in group A-infected macrophages. This effect was abolished by the treatment of group A isolates with PLC inhibitors—D609 and U73122, suggesting the possibility that mycobacterial PLC might induce necrosis in host cells (Assis et al., 2014). Host-derived PLC plays a very important role in mycobacterial infections and pathogenesis (Sukumaran et al., 2003; Bandyopadhaya et al., 2009). Knockdown of host PLC-γ1 using si-RNA caused increased phagocytosis and reduced intracellular survival of both MS and Mtb in J774A1 cells. PLC-γ1-deficient cell also expressed higher levels of tumor necrosis factor-α (TNF-α) and RANTES during Mtb infection. These observations suggest an important role of PLC-γ1 in mycobacterial infection (Paroha et al., 2020). Another study conducted by the same group also reported that the knockdown of host PLC-γ2 in J774A1 cells increased phagocytosis and reduced intracellular survival of Mtb. PLC-γ2-deficient cells also show increased expression of pro-inflammatory cytokines during Mtb infection. In the same study, increased PLC-γ2 phosphorylation during Mtb but not in MS was also reported. These studies suggest that different PLC isoforms may be involved in the regulation of mycobacterial infection (Paroha et al., 2019; Paroha et al., 2020). However, the mechanism of PLC phosphorylation during Mtb infection remains to be determined and represents a promising area of investigation.

**Figure 4:**
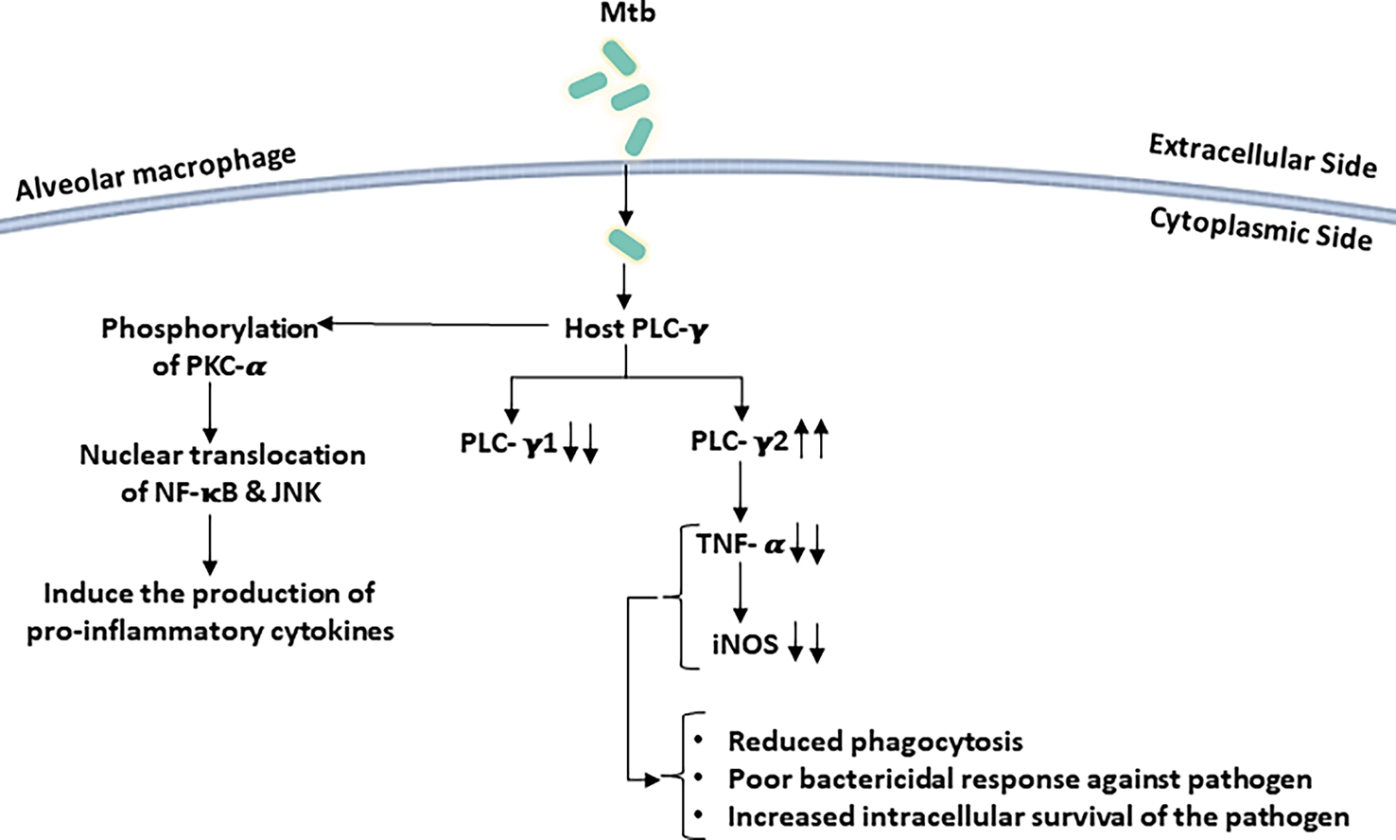
*Mycobacterium tuberculosis* (Mtb) infection perturbs host PLC signaling: infections of macrophage with Mtb selectively activate/deactivate PLC isoforms. PLC-γ2 is activated following Mtb infection while PLC-γ1 expression is reduced in host cells. These perturbations in host PLC signaling result in poor bactericidal responses by macrophages.

It was reported that when human neutrophils were infected with Mtb Ra, an avirulent derivative of Mtb H37Rv, it promoted the production of reactive oxygen intermediate (ROI) through tyrosine phosphorylation of PLC-γ2 by a tyrosine kinase. Inhibition of PLC-γ2 results in reduced ROI production induced by Mtb (Perskvist et al., 2000). To regulate the cytokine production in alveolar macrophages and epithelial cells, transient receptor potential channel-1 (TRPC-1) and its associated endogenous Ca^2+^ channel play an important role. TRPC-1 allows the entry of Ca^2+^ inside the macrophage, which leads to an intense inflammatory response and increases infection susceptibility as compared to the TRPC-1-deficient cells. Activation of PLC-γ in these cells induces the phosphorylation of PKC-α, leading to the translocation of NF-κB and Jun N-terminal protein kinase (JNK) inside the nucleus, resulting in increased production of inflammatory cytokines (**Figure 4**). Thus, an increased level of inflammatory cytokines might provide protection from invading pathogens (Zhou et al., 2015).

After recognition of mycobacteria by macrophages, the secretion of pro-inflammatory cytokines (like TNF-α and RANTES) and chemokines is an important step to mediate the immune response against the bacilli. Treatment with PLC inhibitors showed an inhibitory effect on the secretion of pro-inflammatory cytokines, suggesting that PLC signaling in macrophages might regulate the immune response against the mycobacteria (Yadav et al., 2006; Paroha et al., 2020).

During infection of macrophages by Mtb, the expression of the plc operon in Mtb was significantly upregulated for 24 h of infection (Raynaud et al., 2002). Available literature suggests that the PLC of mycobacterial origin is involved in pathogenicity, but some contrary studies are also reported, and further investigations are required to clearly understand the role of PLC in the virulence of Mtb (Le Chevalier et al., 2015).

### *Listeria monocytogenes* PLC is required to protect pathogens from host autophagy destruction and promote cell-to-cell transmission

*Listeria monocytogenes* (*L. monocytogenes*) is a facultative intracellular gram-positive, rod-shaped pathogen that can cause serious infections in humans and domestic animals (Dabiri et al., 1990; Smith et al., 1995; Songer, 1997; Schlech and Acheson, 2000). This pathogen has a remarkable capability to multiply and spread from cell to cell without exposure to the extracellular environment. To gain entry into host cells, *L. monocytogenes* induces the phagocytic as well as nonphagocytic cells for its internalization (Tattoli et al., 2013).

**Figure 5:**
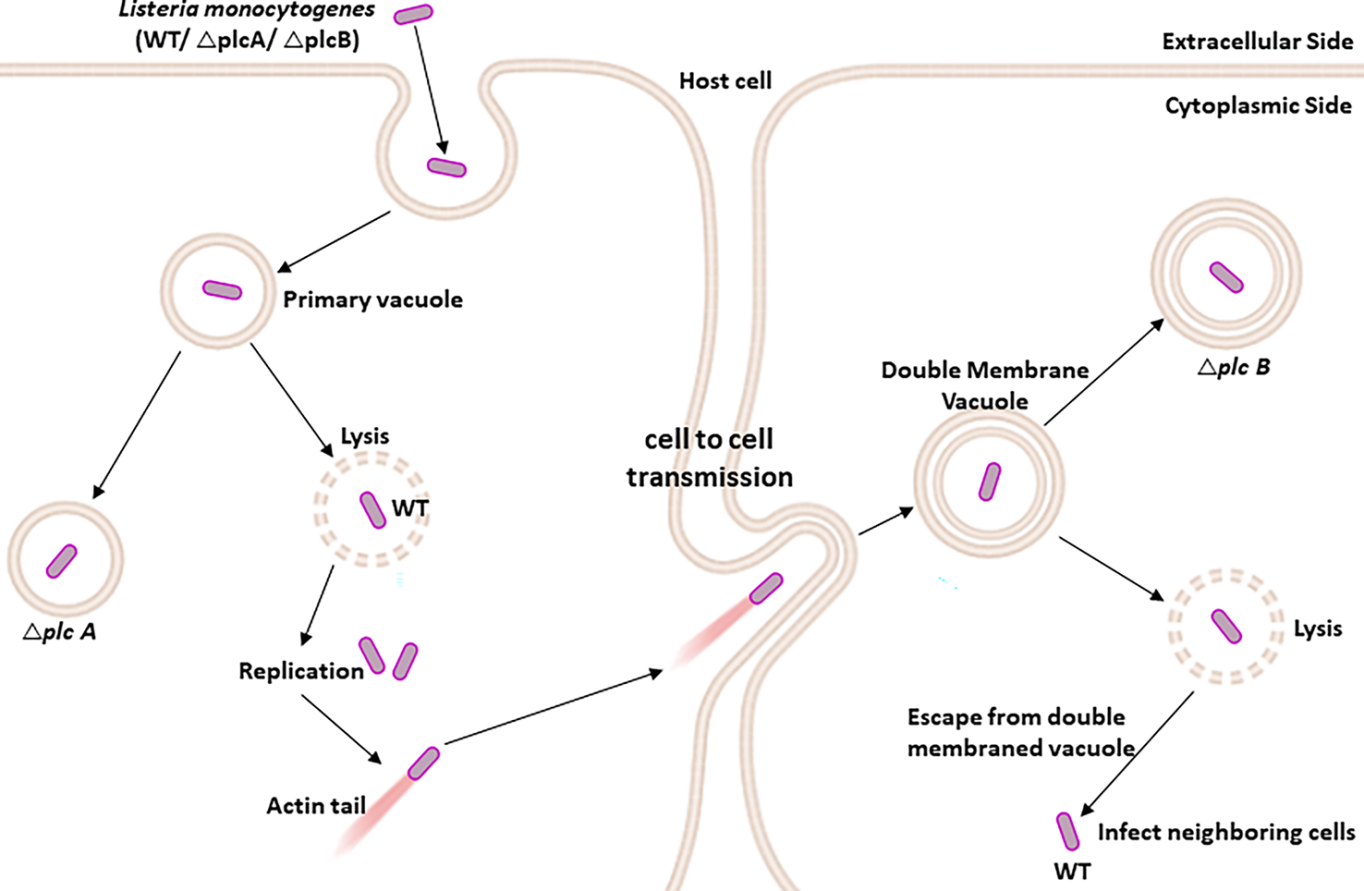
PLC-A and PLC-B promote cell-to-cell spread during *Listeria monocytogenes* infection: After internalization by the host immune cells, *L. monocytogenes* is present inside the primary vacuole. The *L. monocytogenes* (WT) has the ability to lyse the primary vacuole and escape into the host cytosol, where it is free to propel to the host cell surface through the host-specific actin filament and form pseudopod-like structures, allowing them to spread from one cell to another *via* insert forming double-membrane vacuole. Again, the *L. monocytogenes* lyses the double-membrane vacuole to further infect the nearby cells. While the *L. monocytogenes*Δ*plcA* shows an impaired ability to escape from the primary vacuole, *L. monocytogenes* Δ*plcB* shows reduced potential to escape from the double-membrane vacuole.

The practice of living as a pathogen within the host can be divided into six junctures, including (i) the association of bacterium with microvilli on the plasma membrane, (ii) internalization by professional and non-professional phagocytes, (iii) escape from the entry vacuole, (iv) replication in the cytosol, (v) propelled to the cell surface with the help of host actin filaments and form pseudopod-like structure, and (vi) spread through the cell to cell, forming a double-membrane vacuole **(** **Figure 5** **)** (Tilney and Portnoy, 1989; Poussin et al., 2009; Tattoli et al., 2013).

*L. monocytogenes* has evolved to subvert the autophagy flux in the infected cells through PLC activity (Tattoli et al., 2013). PLC produced by *L. monocytogenes* plays a significant role in escaping from the entry vacuole and also helps the pathogen spread from one cell to another cell (Smith et al., 1995). PI-PLC and PC-PLC are encoded by *plcA* and *plcB* genes, respectively (Camilli et al., 1993; Smith et al., 1995; Tattoli et al., 2013). PI-PLC is secreted in an active form, whereas PC-PLC is secreted as an inactive proenzyme in the cytosol of infected host cells and rapidly degraded by the proteasome. PC-PLC is activated by metalloprotease, another enzyme secreted by the pathogen (Marquis et al., 1997).

For the pathogenesis of *L. monocytogenes*, several contiguous genes are reported to be required, which are organized in the following sequence on the regulon: *prfA plcA*, *holy*, *mpl*, *actA*, and *plcB* **(** **Table 1** **)** (Portnoy et al., 1992). PI-PLC has a dual role in pathogenesis. It positively regulates the expression of the *prfA* gene, the product of which regulates the expression of genes imperative for efficient cell-cell spread (Mengaud et al., 1989; Mengaud et al., 1991). PI-PLC is also required for bacterial growth in the murine liver. *In vivo*, the precise molecular events occurring during the action of PI-PLC are not yet clear, but some studies suggest that PI-PLC mediates the systematic lysis of host phagolysosome (Camilli et al., 1993).

**Table 1:** Several genes and their functions are involved during the pathogenesis of *L. monocytogenes*.

Sr. No.	Gene name	Gene function	References
1.	*inlA*	Responsible for the internalization of the bacteria.	(Roche et al., 2009)
2.	*hly*	Encodes the listeriolysin-*O* (pore-forming cytolysin, evolved for activity in a phagosome having low optimum pH) and helps the bacterium to escape from the vacuole.	(Glomski et al., 2002)
3.	*actA*	Surface protein induces the host cell to polymerize the actin filament.	(Roche et al., 2009)
4.	*plcA*	Might help to escape the bacterium from the phagolysosomal vacuole.	(Camilli et al., 1993)
5.	*plcB*	Might help the bacterium to escape from the double-membraned structure and also helps in cell-to-cell spread.	(Roche et al., 2009)
6.	*prfA*	Positive regulatory factor acts as a transcription factor.	(Camilli et al., 1993)
7.	*mpl*	Encodes the metalloprotease, which converts the 33-kDa inactive form of PC-PLC into the 28-kDa active form of PC-PLC.	(Camilli et al., 1993)

Intracellular pathogens like *L. monocytogenes* exploit the host processes to progress pathogenesis in a regulated manner. Depending on the subcellular localization of bacteria, two types of regulation may occur (Marquis et al., 1997). The proteolytic activation of the inactive form of PC-PLC in vacuoles occurs by bacterial proteases, leading to the acidification of the vacuole, whereas the host proteases degrade inactive PC-PLC in the cytosol. As a result of this, PC-PLC shows a short half-life (<15 min) and is essentially present in its inactive form within the cytosol. Interestingly, when the degradation of the inactive form of PC-PLC was inhibited, no mature PC-PLC was detectable in the cytosol (Rock et al., 1994; Marquis et al., 1997. There may be two mechanisms through which the production of active PC-PLC in the cytosol can be prevented: degradation or lack of activation (Marquis et al., 1997). Thus, these observations suggest that the activity of PC-PLC is restricted to the vacuolar compartment (Smith et al., 1995).

Regulated proteolysis and the optimum pH are required by the bacterial virulence factors to easily execute the cell-cell spread, causing the least damage to the host cell otherwise caused by listeriolysin-*O* cytotoxicity (Jones et al., 1996).

Both the phospholipases, plcA and plcB, have overlapping functions in the pathogenesis of *L. monocytogenes* infections. *plcA* mutant showed a slightly reduced capability to escape from the primary vacuole and three-fold less virulence than its wild-type counterpart *plcB* mutant was reported to have decreased potential to escape from the double membraned vacuole structure with 20-fold less virulence and has no measurable defects in escape from a primary vacuole. Indeed, the mutant lacking both PLCs was severely impaired in its ability to escape from the primary vacuoles and cell–cell spread and was 500-fold less virulent (Smith et al., 1995).

Levels of DAG and ceramide were substantially increased in the host cells 4 to 5 h postinfection with *L. monocytogenes*. Such an increase in the levels of DAG and ceramide may be attributed to PLCs derived from pathogens, as the plcA:plcB double mutant of *L. monocytogenes* failed to increase the levels of DAG and ceramide (Smith et al., 1995).

### PLC promotes pathogen colonization and lung inflammation during *Pseudomonas aeruginosa* infection

*Pseudomonas aeruginosa* (*P. aeruginosa*) is a gram-negative, opportunistic pathogen that causes a wide array of acute (sepsis) and chronic infections (pulmonary), including cystic fibrosis (CF), chronic wound infections, and postsurgical infections, especially in immunocompromised individuals (Vasil et al., 2009; Wargo et al., 2011; Wu et al., 2015; Diggle and Whiteley, 2020). PLCs contributed to multiple aspects of *P. aeruginosa* pathogenesis (Berk et al., 1987). *P. aeruginosa* expresses three extracellular PLCs: the hemolytic PLC known as PlcH and the nonhemolytic PlcN and PlcB (Ostroff and Vasil, 1987; Barker et al., 2004; Wargo et al., 2009; Klockgether and Tümmler, 2017). While PlcH and PlcN are secreted through the twin-arginine translocase (TAT) secretory system, PlcB is secreted through the Sec pathway (Ochsner et al., 2002; Barker et al., 2004; Snyder et al., 2006). All the PLCs from *P. aeruginosa* can cleave PC, but only PlcB can hydrolyze phosphatidylethanolamine (Esselmann and Liu, 1961; Ochsner et al., 2002; Barker et al., 2004; Snyder et al., 2006).

The heterodimeric complex of novel PlcHR consists of two subunits: PlcH as the active center and PlcR as a chaperone protein that modulates the enzymatic activity and is required for the PlcH secretion itself (Stonehouse et al., 2002).

The PLC of *P. aeruginosa* has been shown to induce aggregation in platelets derived from platelet-rich human plasma in a concentration-dependent manner, and the enzymatic activity of PLC was required for platelet aggregation (Coutinho et al., 1988). Thus, PLC is heat-labile by nature (Berka et al., 1981). Some studies suggest that *P. aeruginosa* may have two hemolytic virulence factors that might cause paralysis, dermonecrosis, footpad swelling, vascular permeability, and death in mice (Berk et al., 1987).

Inflammation is one of the hallmark features of pulmonary infections caused by *P. aeruginosa* (Titball, 1998). PlcH has been shown to induce an inflammatory response in human neutrophils and polymorphonuclear cells *in vitro*. Treatment of neutrophils with PlcH but not PlcN increased the production of inflammation markers such as oxygen metabolites, leukotriene-B4 (LTB4), and histamine (König et al., 1997). Furthermore, PLC from *P. aeruginosa* induced an inflammatory response in mice in an enzyme activity-dependent manner. Intraperitoneal injection of PLC resulted in the induction of an inflammatory response in mice characterized by the accumulation of plasma proteins, inflammatory cells, and arachidonic acid metabolites such as LTB4, LTC4, LTD4, prostaglandin E2 (PGE2), PGF2-alpha, and thromboxane B2 (Meyers and Berk, 1990). While LTB4 acts as a strong stimulator for chemotactic aggregation of granulocytes, leukotriene-C4 and leukotriene-D4 cause the wheal and flare response (Camp et al., 1983; Meyers and Berk, 1990). PGE2 is known to be a powerful vasodilator believed to cause edema, erythema, and pain (Williams, 1979). Granström et al. demonstrated that the level of PLC antibodies was elevated in CF patients chronically infected with *P. aeruginosa*, suggesting that it helps in chronic infection (Granström et al, 1984). Several *in vitro* studies with human bronchial cells suggest a role for *P. aeruginosa* PLC in exacerbated inflammation in CF lungs, which is responsible for the poor prognosis (Sener et al., 1999; Wargo et al., 2011). There is a scientific consensus that neutrophils and pulmonary macrophages play a pivotal role in clearing pathogenic bacterial infections from the human host (Sener et al.,1999). Despite the abundance of neutrophils in the lung tissues, PlcHR helps *P. aeruginosa* survive in such detrimental conditions by suppressing respiratory neutrophil burst response (Terada et al., 1999). Other than playing a role in inducing an inflammatory response in the lungs, PLC may also contribute to lung function by destroying lung surfactants. The PlcH of *P. aeruginosa* has been reported to act as a destructive determinant for lung surfactant. The wild-type strain of *P. aeruginosa* significantly impairs the functioning of the lung surfactants, whereas the PlcHR mutant strain is remarkably less potent to cause infection (Montes et al., 2007). PLC helps hydrolyze PC, an abundant phospholipid in the lung surfactant, which can then be converted to glycine betaine. The accumulation of glycine betaine within the bacterial cell is thought to protect against the high osmolarity conditions found in the lung tissue, further helping bacteria to colonize the lung (Shortridge et al.,1992; Fitzsimmons et al., 2012). PLC also plays a role in biofilm formation *in vitro* and in animal models, further suggesting its central role in *P. aeruginosa*-associated morbidity and mortality in CF patients (Wargo et al., 2011; Jackson et al., 2013).

PlcH also contributes to *P. aeruginosa* virulence by stimulating the activity of the anaerobic respirational regulator Anr by releasing choline (Fitzsimmons et al., 2012; Jackson et al., 2013). Microarray analysis revealed that the nonfunctional form of PlcH had decreased levels of Anr-regulated transcripts compared with that of the wild-type strain. PlcH promotes the Anr activity, which contributes to the severity of the disease even in acute phase infection, where the oxygen level is believed to be high. Thus, Anr’s activity promotes colonization in the host (Jackson et al., 2013). An increase in Anr activity activates numerous pathways involved in *P. aeruginosa* virulence, respiration, and biofilm formation. The *anr* mutant was severely impaired in its ability to colonize lung tissue and was cleared from the lung much faster compared to the wild-type strain (Yoon et al., 2002; Worlitzsch et al., 2002). These findings suggest that Anr could be a good target for developing new therapies. Other pathogens have also been reported to require Anr homologs for their virulence (Baltes et al., 2005; Mattoo et al.,2004; Bartolini et al., 2006; Marteyn et al., 2010). This way, it is also possible to devise some beneficial approaches for the treatment of other diseases.

Angiogenesis (formation of blood vessels) helps in the wound healing process, in which endothelial cells play a significant role. PlcHR2 has been reported as a factor that decisively induces cytotoxicity in endothelial cells, affecting angiogenesis (Vasil et al., 2009).

### PLC induces hemolysis and apoptosis in host cells during *Helicobacter pylori* infection

*Helicobacter pylori* (*H. pylori*) is a spiral-shaped, gram-negative, microaerophilic bacteria that colonizes the gastric mucosa of humans (Warren and Marshall, 1983; Brown, 2000; Atherton, 2006). Initially, *H. pylori* was classified under *Campylobacter*; later, in 1989, it was classified under a new genus called *Helicobacter* and was named *Helicobacter pylori* (Goodwin et al., 1989). It was recognized as the common causative agent of chronic gastritis and duodenal ulcers (Warren and Marshall, 1983; Hemalatha et al., 1991; Marshall and Warren et al., 1984). *H. pylori* adheres to the antrum’s mucosal lining and releases urease to protect itself from the acidic environment (Hemalatha et al., 1991; Nakshabendi et al., 1996).

Several phospholipases are reported to be produced by *H. pylori*, including PLC, which might help the bacteria to cause ulcerogenic activity. *H. pylori*-mediated hemolysis might be related to their capability for the production of PLC. Studies conducted on erythrocytes derived from humans, horses, sheep, and guinea pigs showed variable degrees of hemolytic activity induced by *H. pylori*. It was also reported that the strains isolated from the patients with duodenal ulcers showed a higher efficiency in producing elevated levels of PLC compared to those with gastritis (Wetherall and Johnson et al., 1989; Daw et al., 1994). These observations suggest that PLC activity might be required for hemolytic activity and virulence during the pathogenicity of *H. pylori*.

**Figure 6:**
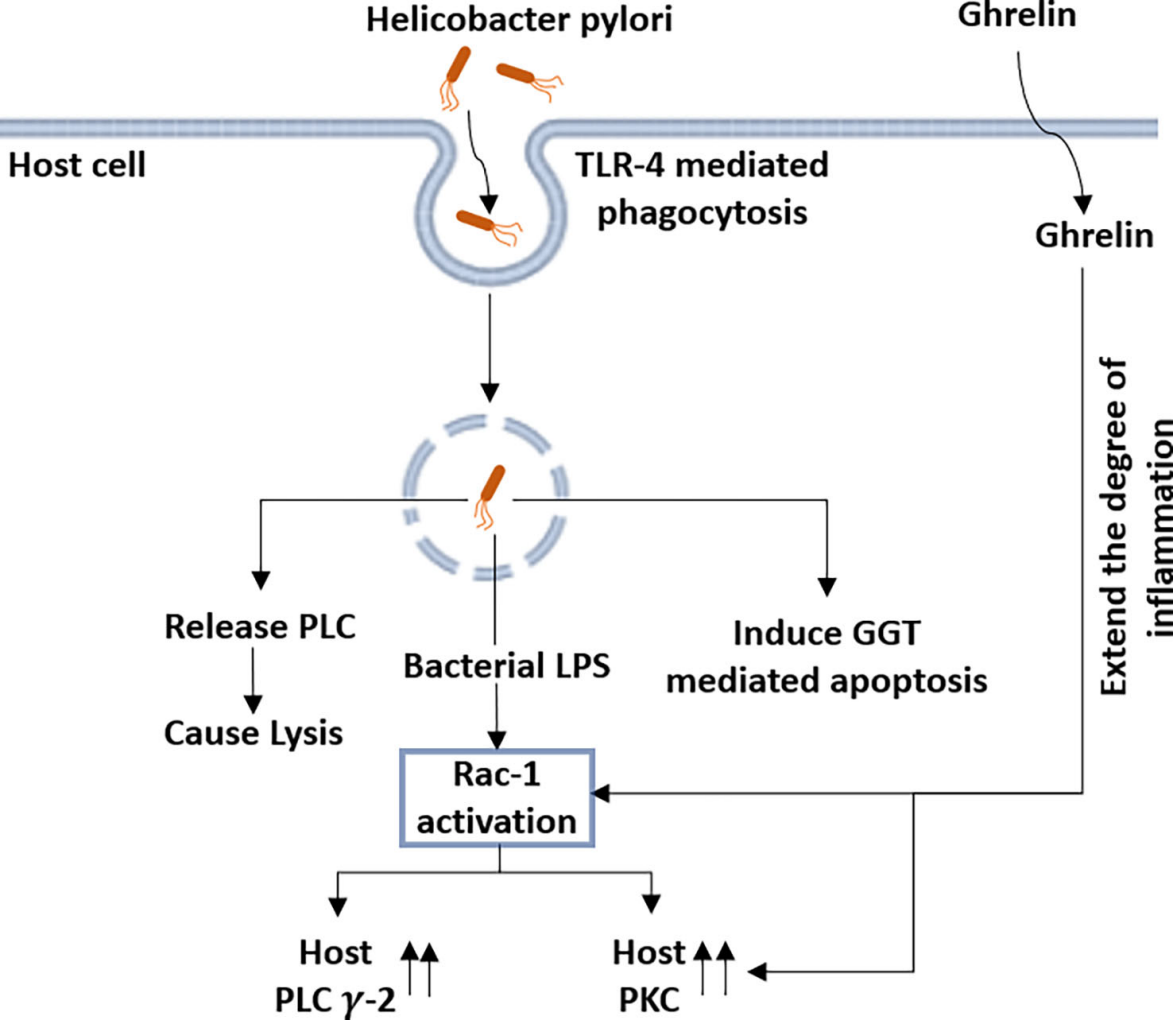
*Helicobacter pylori* (*H. pylori*) infection modulates the expression of host-derived PLC-γ2 along with several other host proteins: following the toll-like receptor 4 (TLR-4)-mediated phagocytosis by host cells, *H. pylori* releases the PLC in the cytosol, causing lysis of the host cells. *H. pylori* also induces the γ-glutamyl transpeptidase (GGT)-mediated apoptosis of the host cell. Lipopolysaccharide (LPS) from *H. pylori* activates the Rac-1, which increases the expression of both host-specific PLC-γ2 and PKC. Ghrelin further increases PKC activity and also has a modulatory effect on Rac-1 activation, which leads to inflammation.

Lipopolysaccharide (LPS) from *H. pylori* has been recognized as a potent virulence factor, causing an inflammatory response in mucosal epithelial cells, leading to gastritis and duodenal ulcers (Piotrowski et al., 1997; Rieder et al., 2003). Ghrelin, a peptide hormone, is an endogenous factor that regulates the intensity of gastric mucosal inflammation in response to *H. pylori* infection (Kojima et al., 1999; Osawa et al., 2005; Waseem et al., 2008). Both, the LPS of the bacterium and the ghrelin of the host rely on the activation of different signaling pathways involving PI-PLC in order to exert their effects the gastric mucosal inflammation **(** **Figure 6** **)** (Slomiany and Slomiany, 2015a).

Following toll-like receptor 4 (TLR4)-mediated phagocytosis of the bacterium, LPS from *H. pylori* induces the activation of Rac1 and causes gastric mucosal inflammation (Kong and Gee, 2008; Slomiany and Slomiany, 2015b). It has been reported that Rac1 activation significantly increases the activities of host-specific PLC-γ2 and PKC and the increase in ghrelin further increases PKC activity. Slomiany et al. reported that activation of host PLC-γ2 during infection with *H. pylori* is dependent on rac1 and is modulated by the ghrelin hormone **(** **Figure 6** **)** (Slomiany and Slomiany, 2015b).

Infection with *H. pylori* in normal human gastric mucous epithelial cells alters the intracellular Ca^2+^ levels in PLC-dependent manner (Marlink et al., 2003). Infection of gastric adenocarcinoma cells with *H. pylori* induces apoptosis in these cells. Apoptosis was shown to be associated with an increase in intracellular Ca^2+^. An increase in intracellular Ca^2+^ levels and induction of apoptosis by g-glutamyl transpeptidase (GGT) was abolished with the treatment of a PLC inhibitor (U71322) and IP3 antagonist (xestospongin). These observations suggest the requirement of PLC in *H. pylori*-induced apoptosis in the host cell (Park et al., 2014; Ricci et al., 2014; Boonyanugomol et al., 2012). Studies discussed above strongly establish PI-PLC as an important virulence factor in the pathogenesis of *H. pylori*.

### PLCs in other bacterial infections

Different PLC isoforms are also reported to play crucial roles during several other bacterial infections, such as those caused by *Legionella pneumophila* (*L. pneumophila*), *Staphylococcus aureus* (*S. aureus*), *Burkholderia cepacia* (*B. cepacia*)*, and* reproductive tract-associated bacterial infections. *L. pneumophila* is a gram-negative bacillus and the causative agent of Legionnaires. This pathogen contains three PLC (PLC-A, PLC-B, and PLC-C) genes identified in its genome. These PLCs are reported to be released inside the host cell through the secretory systems, where they manipulate host transcription, translation, phospholipid pool, and vesicle trafficking pathways. Furthermore, these PLCs also influence the host phagosome maturation and cell death pathways (Hiller et al., 2018). The role of PLCs in Legionnaires is not yet clearly understood and requires further investigation. *S. aureus* has been reported to release PI-PLC with the ability to hydrolyze the PI and glucosyl phosphatidyl inositol (GPI)-linked proteins present on the host cell surface (Daugherty and Low, 1993). These PI-PLCs are involved in the regulation of accessory gene regulator quorum sensing systems. The overexpression of secreted PI-PLC in *S. aureus* caused more severe disease, suggesting its role as a virulence factor. In line with these observations, a PLC mutant of *S. aureus* exhibits defective survival in human blood and neutrophils suggesting that PI-PLC might contribute to the survival of *S. aureus* in the host (White et al., 2014). Two PLC isoforms have been identified in *B. cepacia*, one with hemolytic (PLC-H) activity and the other without hemolytic (PLC-N) activity (Weingart and Hooke, 1999a; 1999b). In *Bacillus anthracis*, three PLC genes have been identified, which are homologous to the PLCs from *L. monocytogenes*. The functions of these PLCs are not understood so far, but it is speculated that these PLCs might act as virulence factors (Heffernan et al., 2006; Heffernan et al., 2007). Several aerobic and anaerobic bacteria associated with reproductive tract infection show PLC activities, which may be linked directly or indirectly with localized cells/tissue damage and contribute to the virulence of the microorganisms (McGregor et al., 1991).

## Conclusion

Phospholipids have both hydrophilic and hydrophobic domains and function as the main constituent of all biological membranes. Cell surface-specific receptors receive extracellular stimuli and transmit them inside the cell in order to produce cellular effects. Thirteen different PLC isoforms with unique structures mediate the diverse signaling functions. Several extracellular signals activate the PLC enzyme, resulting in the cleavage of phospholipids into the bi-products, which in turn modulate the activities of various targets to contribute to context-specific cellular functions. PLC plays a crucial role in the pathogenesis of several bacteria and exerts a variety of effects that contribute to the disease-specific perturbation in host cells. The PLC enzyme as a virulence factor needs to be further investigated in other bacterial infections. It is important to understand how the cells discriminate between the signals and activate signal-specific PLC/PLCs whether the PLC isoforms work independently or work in a coordinated manner. Some chemical inhibitors are used to inhibit PLC’s functions. Several studies reported that 1-[6-(17b-3-methoxyestra-1,3,5 (10)trien-17-yl)amino)hexyl]-1H-pyrrole-2,5-dione (U71322) blocks the recruitment of PLC to the membrane and may be used as a pan PLC inhibitor. Klein et al. reported that the U71322 directly activates the PLC-γ1, PLC-β2, and PLC-β3 of humans instead of inhibiting them (Klein et al., 2011). Some other inhibitors, like 2-nitro-4-carboxyphenyl *N*,*N*-diphenylcarbamate (NCDC) and 1-*O*-octadecyl-2-*O*-methyl-rac-glycero-3-phosphorylcholine (ET-18-OCH3), are also used to inhibit PLCs. Inhibiting PLCs with different approaches, including chemical inhibitors, may be a promising strategy to manipulate the outcome of bacterial infections.

## Author contributions

VS, RR, and BM did literature search and wrote the manuscript. RC, AS, and AK gave conceptual inputs and edited the manuscript as per their specializations in the field. SC conceptualized the theme of the manuscript, supervised the overall preparation of the manuscript, and contributed to the writing and editing of the final version of the manuscript. All authors contributed to the article and approved the submitted version.

## Glossary

**Table:**

Anr	anaerobic respiration
Asn^294^	asparagine residue at 294^th^ position
*B. cepacia*	*Burkholderia cepacia*
*C. perfringens*	*Clostridium perfringens*
Ca^2+^	calcium ion
CF	cystic fibrosis
COX-2	cyclooxygenase-2
DAG	di-acyl-glycerol
EP-2	prostaglandin E2 receptor-2
EP-4	prostaglandin E2 receptor-4
ET-18-OCH3	1-*O*-octadecyl-2-*O*-methyl-rac-glycero-3-phosphorylcholine
GEF	guanine exchange factor
GGT	λ-glutamyl transpeptidase
GPI	glucosyl phosphatidyl inositol
*H. pylori*	*Helicobacter pylori*
His^311^	histidine residue at 311th position
His^356^	histidine residue at 356th position
JNK	Jun N-terminal protein kinase
*L. monocytogenes*	*Listeria monocytogenes*
*L. pneumophila*	*Legionella pneumophila*
LPS	lipopolysaccharide
LTB4	leukotrine B4
MAPK	mitogen activated protein kinases
MS	*Mycobacterium smegmatis*
Mtb	*Mycobacterium tuberculosis*
NCDC	*N*,*N*-diphenylcarbamate
NFκB	nuclear factor-κB
NO	nitric oxide
O_2_	superoxide anions
IP3	inositol triphosphate
*P. aeruginosa*	*Pseudomonas aeruginosa*
PC	phosphatidylcholine
PDK 1	3-phosphoinositide-dependent protein kinase 1
PI	phosphatidylinositol
PIP2	phosphatidylinositol 4,5 bisphosphate
PKC	protein kinase C
PL	phospholipase
PLA	phospholipase A
PLB	phospholipase B
PLC	phospholipase C
PLD	phospholipase D
PH domain	Pleckstrin homology domain
*S. aureus*	*Staphylococcus aureus*
TNF-α	tumor necrosis factor-α
TLR	Toll-like receptor
U71322	1-[6-((17β-3-methoxyestra-1,3,5(10)trien-17 yl)amino)hexyl]-1H-pyrrole-2,5-dione
Rac 1	Ras-related C3 botulinum toxin substrate 1
ROI	reactive oxygen intermediates
SH2 domain	Src homology 2 domain
SH3 domain	Src homology 3 domain
TAT	Twin arginine translocase
TB	tuberculosis
TIM	triose phosphate isomerase
TrkA	tropomyosin receptor kinase A
TRPC-1	transient receptor potential channel 1
Tyr^62^	tyrosine residue at 62nd position
WT	wild type
Zn^2+^	zinc ion

## References

[B1] Abdulnour R. E. Howrylak J. A. Tavares A. H. Douda D. N. Henkels K. M. Miller T. E.. (2018). Phospholipase D isoforms differentially regulate leukocyte responses to acute lung injury. J. Leukoc. Biol. 103 (5), 919–932. doi: 10.1002/JLB.3A0617-252RR 29437245 PMC6375305

[B2] Alape-Girón A. Flores-Díaz M. Guillouard I. Naylor C. E. Titball R. W. Rucavado A.. (2000). Identification of residues critical for toxicity in *Clostridium perfringens* phospholipase C, the key toxin in gas gangrene. Eur. J. Biochem. 267 (16), 5191–5197. doi: 10.1046/j.1432-1327.2000.01588.x 10931204

[B3] Allen V. Swigart P. Cheung R. Cockcroft S. Katan M. (1997). Regulation of inositol lipid-specific phospholipase Cδ by changes in Ca2+ ion concentrations. Biochem. J. 327 (Pt 2), 545–552. doi: 10.1042/bj3270545 9359428 PMC1218828

[B4] Aloulou A. Rahier R. Arhab Y. Noiriel A. Abousalham A. (2018). Phospholipases: An overview. In Sandoval G. ed. Lipases and Phospholipases: Methods and Protocols. Methods in Molecular Biology. New York, NY: Springer New York. 10.1007/978-1-4939-8672-9_3 30109646

[B5] Assis P. A. Espíndola M. S. Paula-Silva F. W. Rios W. M. Pereira P. A. Leão S. C.. (2014). *Mycobacterium tuberculosis* expressing phospholipase C subverts PGE2 synthesis and induces necrosis in alveolar macrophages. BMC Microbiol. 14, 128. doi: 10.1186/1471-2180-14-128 24886263 PMC4057917

[B6] Atherton J. C. (2006). The pathogenesis of *Helicobacter pylori*-induced gastro-duodenal diseases. Annu. Rev. Pathol. 1, 63–96. doi: 10.1146/annurev.pathol.1.110304.100125 18039108

[B7] Aurass P. Schlegel M. Metwally O. Harding C.R. Schroeder G.N. Frankel G. (2013). The Legionella pneumophila Dot/Icm-secreted Effector PlcC/CegC1 Together with PlcA and PlcB Promotes Virulence and Belongs to a Novel Zinc Metallophospholipase C Family Present in Bacteria and Fungi. J Biol Chem 288 (16), 11080–11092. doi: 10.1074/jbc.M112.426049 23457299 PMC3630882

[B8] Awad M. M. Bryant A. E. Stevens D. L. Rood J. I. (1995). Virulence studies on chromosomal alpha-toxin and theta-toxin mutants constructed by allelic exchange provide genetic evidence for the essential role of alpha-toxin in *Clostridium perfringens*-mediated gas gangrene. Mol. Microbiol. 15 (2), 191–202. doi: 10.1111/j.1365-2958.1995.tb02234.x 7746141

[B9] Bakala N'goma J. C. Schué M. Carrière F. Geerlof A. Canaan S. (2010). Evidence for the cytotoxic effects of *Mycobacterium tuberculosis* phospholipase C towards macrophages. Biochim. Biophys. Acta 1801 (12), 1305–1313. doi: 10.1016/j.bbalip.2010.08.007 20736081

[B10] Baltes N. N'diaye M. Jacobsen I. D. Maas A. Buettner F. F. Gerlach G. F. (2005). Deletion of the anaerobic regulator HlyX causes reduced colonization and persistence of *Actinobacillus pleuropneumoniae* in the porcine respiratory tract. Infect. Immun. 73 (8), 4614–4619. doi: 10.1128/IAI.73.8.4614-4619.2005 16040973 PMC1201192

[B11] Bandyopadhaya A. Das D. Chaudhuri K. (2009). Involvement of intracellular signaling cascades in inflammatory responses in human intestinal epithelial cells following *Vibrio cholerae* infection. Mol. Immunol. 46 (6), 1129–1139. doi: 10.1016/j.molimm.2008.11.003 19110311

[B12] Barker A. P. Vasil A. I. Filloux A. Ball G. Wilderman P. J. Vasil M. L. (2004). A novel extracellular phospholipase C of *Pseudomonas aeruginosa* is required for phospholipid chemotaxis. Mol. Microbiol. 53 (4), 1089–1098. doi: 10.1111/j.1365-2958.2004.04189.x 15306013

[B13] Barrett-Bee K. Hayes Y. Wilson R. G. Ryley J. F. (1985). A comparison of phospholipase activity, cellular adherence and pathogenicity of yeasts. J. Gen. Microbiol. 131 (5), 1217–1221. doi: 10.1099/00221287-131-5-1217 3894572

[B14] Bar-Sagi D. Rotin D. Batzer A. Mandiyan V. Schlessinger J. (1993). SH3 domains direct cellular localization of signaling molecules. Cell 74 (1), 83–91. doi: 10.1016/0092-8674(93)90296-3 8334708

[B15] Bartolini E. Frigimelica E. Giovinazzi S. Galli G. Shaik Y. Genco C.. (2006). Role of FNR and FNR-regulated, sugar fermentation genes in *Neisseria meningitidis* infection. Mol. Microbiol. 60 (4), 963–972. doi: 10.1111/j.1365-2958.2006.05163.x 16677307 PMC2258229

[B16] Berk R. S. Brown D. Coutinho I. Meyers D. (1987). *In vivo* studies with two phospholipase C fractions from *Pseudomonas aeruginosa* . Infect. Immun. 55 (7), 1728–1730. doi: 10.1128/iai.55.7.1728-1730.1987 3110070 PMC260587

[B17] Berka R. M. Gray G. L. Vasil M. L. (1981). Studies of phospholipase C (heat-labile hemolysin) in *Pseudomonas aeruginosa* . Infect. Immun. 34 (3), 1071–1074. doi: 10.1128/iai.34.3.1071-1074.1981 6800952 PMC350978

[B18] Bonvini E. DeBell K. E. Verí M. C. Graham L. Stoica B. Laborda J.. (2003). On the mechanism coupling phospholipase Cγ1 to the b- and T-cell antigen receptors. Adv. Enzyme Regul. 43, 245–269. doi: 10.1016/s0065-2571(02)00033-x 12791395

[B19] Boonyanugomol W. Chomvarin C. Song J. Y. Kim K. M. Kim J. M. Cho M. J.. (2012). Effects of *Helicobacter pylori* γ-glutamyltranspeptidase on apoptosis and inflammation in human biliary cells. Dig Dis. Sci. 57 (10), 2615–2624. doi: 10.1007/s10620-012-2216-2 22581342

[B20] Brown L. M. (2000). *Helicobacter pylori*: epidemiology and routes of transmission. Epidemiol. Rev. 22 (2), 283–297. doi: 10.1093/oxfordjournals.epirev.a018040 11218379

[B21] Bunney T. D. Katan M. (2011). PLC Regulation: emerging pictures for molecular mechanisms. Trends Biochem. Sci. 36 (2), 88–96. doi: 10.1016/j.tibs.2010.08.003 20870410

[B22] Camilli A. Tilney L. G. Portnoy D. A. (1993). Dual roles of *plcA* in *Listeria monocytogenes* pathogenesis. Mol. Microbiol. 8 (1), 143–157. doi: 10.1111/j.1365-2958.1993.tb01211.x 8388529 PMC4836944

[B23] Camp R. D. Coutts A. A. Greaves M. W. Kay A. B. Walport M. J. (1983). Responses of human skin to intradermal injection of leukotrienes C4, D4 and B4. Br. J. Pharmacol. 80 (3), 497–502. doi: 10.1111/j.1476-5381.1983.tb10721.x 6315118 PMC2044991

[B24] Côtes K. Bakala N'goma J. C. Dhouib R. Douchet I. Maurin D. Carrière F.. (2008). Lipolytic enzymes in *Mycobacterium tuberculosis* . Appl. Microbiol. Biotechnol. 78 (5), 741–749. doi: 10.1007/s00253-008-1397-2 18309478

[B25] Coutinho I. R. Berk R. S. Mammen E. (1988). Platelet aggregation by a phospholipase C from *Pseudomonas aeruginosa* . Thromb. Res. 51 (5), 495–505. doi: 10.1016/0049-3848(88)90115-6 3140410

[B26] Cox L. J. Larman M. G. Saunders C. M. Hashimoto K. Swann K. Lai F. A. (2002). Sperm phospholipase Czeta from humans and cynomolgus monkeys triggers Ca2+ oscillations, activation and development of mouse oocytes. Reproduction 124 (5), 611–623. doi: 10.1530/rep.0.1240611 12416999

[B27] Cox G. M. McDade H. C. Chen S. C. Tucker S. C. Gottfredsson M. Wright L. C.. (2001). Extracellular phospholipase activity is a virulence factor for *Cryptococcus neoformans* . Mol. Microbiol. 39 (1), 166–175. doi: 10.1046/j.1365-2958.2001.02236.x 11123698

[B28] Dabiri G. A. Sanger J. M. Portnoy D. A. Southwick F. S. (1990). *Listeria monocytogenes* moves rapidly through the host-cell cytoplasm by inducing directional actin assembly. Proc. Natl. Acad. Sci. U. S. A. 87 (16), 6068–6072. doi: 10.1073/pnas.87.16.6068 2117270 PMC54473

[B29] Daugherty S. Low M. G. (1993). Cloning, expression, and mutagenesis of phosphatidylinositol-specific phospholipase C from *Staphylococcus aureus*: A potential *Staphylococcal virulence* factor. Infect. Immun. 61 (12), 5078–5089. doi: 10.1128/iai.61.12.5078-5089.1993 8225585 PMC281286

[B30] Daw M. A. Xia H. X. O’Morain C. (1994). *Helicobacter pylori*: phospholipase C and Haemolysis. In Gasbarrini G. Pretolani S. eds. Basic and Clinical Aspects of Helicobacter pylori Infection. Berlin, Heidelberg: Springer, 85–89. doi: 10.1007/978-3-642-78231-2_17

[B31] Diggle S. P. Whiteley M. (2020). Microbe profile: *Pseudomonas aeruginosa*: opportunistic pathogen and lab rat. Microbiol. (Reading). 166 (1), 30–33. doi: 10.1099/mic.0.000860 PMC7273324 31597590

[B32] Ellis MV U. S. Katan M. (1995). Mutations within a highly conserved sequence present in the X region of phosphoinositide-specific phospholipase C-δ 1. Biochem. J. 307 (Pt 1), 69–75. doi: 10.1042/bj3070069 7717996 PMC1136746

[B33] Esselmann M. T. Liu P.V. (1961). Lecithinase production by gram-negative bacteria. J. Bacteriol. 81 (6), 939–945. doi: 10.1128/jb.81.6.939-945.1961 13697414 PMC314763

[B34] Essen L. O. Perisic O. Cheung R. Katan M. Williams R. L. (1996). Crystal structure of a mammalian phosphoinositide-specific phospholipase C δ. Nature 380 (6575), 595–602. doi: 10.1038/380595a0 8602259

[B35] Essen L. O. Perisic O. Lynch D. E. Katan M. Williams R. L. (1997). A ternary metal binding site in the C2 domain of phosphoinositide-specific phospholipase C-δ1. Biochemistry 36 (10), 2753–2762. doi: 10.1021/bi962466t 9062102

[B36] Exton J. H. (1990). Signaling through phosphatidylcholine breakdown. J. Biol. Chem. 265 (1), 1–4. doi: 10.1016/S0021-9258(19)40184-1 2104616

[B37] Falkinham J. O. 3rd. (1996). Epidemiology of infection by nontuberculous mycobacteria. Clin. Microbiol. Rev. 9 (2), 177–215. doi: 10.1128/CMR.9.2.177 8964035 PMC172890

[B38] Fanning A. S. Anderson J. M. (1996). Protein-protein interactions: PDZ domain networks. Curr. Biol. 6 (11), 1385–1388. doi: 10.1016/s0960-9822(96)00737-3 8939589

[B39] Fitzsimmons L. F. Hampel K. J. Wargo M. J. (2012). Cellular choline and glycine betaine pools impact osmoprotection and phospholipase C production in *Pseudomonas aeruginosa* . J. Bacteriol. 194 (17), 4718–4726. doi: 10.1128/JB.00596-12 22753069 PMC3415529

[B40] Flores-Díaz M. Alape-Girón A. (2003). Role of *Clostridium perfringens* phospholipase C in the pathogenesis of gas gangrene. Toxicon 42 (8), 979–986. doi: 10.1016/j.toxicon.2003.11.013 15019495

[B41] Flores-Díaz M. Alape-Girón A. Clark G. Catimel B. Hirabayashi Y. Nice E.. (2005). A cellular deficiency of gangliosides causes hypersensitivity to *Clostridium perfringens* phospholipase C. J. Biol. Chem. 280 (29), 26680–26689. doi: 10.1074/jbc.M500278200 15919667

[B42] Flores-Díaz M. Alape-Girón A. Titball R. W. Moos M. Guillouard I. Cole S.. (1998). UDP-Glucose deficiency causes hypersensitivity to the cytotoxic effect of *Clostridium perfringens* phospholipase C. J. Biol. Chem. 273 (38), 24433–24438. doi: 10.1074/jbc.273.38.24433 9733734

[B43] Flores-Díaz M. Thelestam M. Clark G. C. Titball R. W. Alape-Girón A. (2004). Effects of *Clostridium perfringens* phospholipase C in mammalian cells. Anaerobe 10 (2), 115–123. doi: 10.1016/j.anaerobe.2003.11.002 16701508

[B44] Fujimoto S. Yoshida N. Fukui T. Amanai M. Isobe T. Itagaki C.. (2004). Mammalian phospholipase Cζ induces oocyte activation from the sperm perinuclear matrix. Dev. Biol. 274 (2), 370–383. doi: 10.1016/j.ydbio.2004.07.025 15385165

[B45] Fukami K. Inanobe S. Kanemaru K. Nakamura Y. (2010). Phospholipase C is a key enzyme regulating intracellular calcium and modulating the phosphoinositide balance. Prog. Lipid Res. 49 (4), 429–437. doi: 10.1016/j.plipres.2010.06.001 20553968

[B46] Fukami K. Nakao K. Inoue T. Kataoka Y. Kurokawa M. Fissore R. A.. (2001). Requirement of phospholipase Cδ4 for the zona pellucida-induced acrosome reaction. Science 292 (5518), 920–923. doi: 10.1126/science.1059042 11340203

[B47] Fukami K. Yoshida M. Inoue T. Kurokawa M. Fissore R. A. Yoshida N.. (2003). Phospholipase Cδ4 is required for Ca2+ mobilization essential for acrosome reaction in sperm. J. Cell Biol. 161 (1), 79–88. doi: 10.1083/jcb.200210057 12695499 PMC2172882

[B48] Gifford J. L. Walsh M. P. Vogel H. J. (2007). Structures and metal-ion-binding properties of the Ca2+-binding helix-loop-helix EF-hand motifs. Biochem. J. 405 (2), 199–221. doi: 10.1042/BJ20070255 17590154

[B49] Glomski I. J. Gedde M. M. Tsang A. W. Swanson J. A. Portnoy D. A. (2002). The *Listeria monocytogenes* hemolysin has an acidic pH optimum to compartmentalize activity and prevent damage to infected host cells. J. Cell Biol. 156 (6), 1029–1038. doi: 10.1083/jcb.200201081 11901168 PMC2173464

[B50] Goodwin C. S. Armstrong J. A. Chilvers T. Peters M. Collins M. D. Sly L. (1989). Transfer of *Campylobacter pylori* and Campylobacter mustelae to *Helicobacter* gen. nov. as *Helicobacter pylori* comb. nov. and *Helicobacter mustelae* comb. nov., Respectively. Int J Syst Bacteriol 39 (4), 397–405. doi: 10.1099/00207713-39-4-397

[B51] Gordon S. V. Parish T. (2018). Microbe profile: *Mycobacterium tuberculosis*: Humanity's deadly microbial foe. Microbiol. (Reading). 164 (4), 437–439. doi: 10.1099/mic.0.000601 29465344

[B52] Gout I. Dhand R. Hiles I. D. Fry M. J. Panayotou G. Das P.. (1993). The GTPase dynamin binds to and is activated by a subset of SH3 domains. Cell 75 (1), 25–36. doi: 10.1016/S0092-8674(05)80081-9 8402898

[B53] Granström M. Ericsson A. Strandvik B. Wretlind B. Pavlovskis O. R. Berka R.. (1984). Relation between antibody response to *Pseudomonas aeruginosa* exoproteins and colonization/infection in patients with cystic fibrosis. Acta Paediatr. Scand. 73 (6), 772–777. doi: 10.1111/j.1651-2227.1984.tb17774.x 6441448

[B54] Gustafson C. Tagesson C. (1990). Phospholipase C from *Clostridium perfringens* stimulates phospholipase A2-mediated arachidonic acid release in cultured intestinal epithelial cells (INT 407). Scand. J. Gastroenterol. 25 (4), 363–371. doi: 10.3109/00365529009095500 2110684

[B55] Harlan J. E. Hajduk P. J. Yoon H. S. Fesik S. W. (1994). Pleckstrin homology domains bind to phosphatidylinositol-4,5-bisphosphate. Nature 371 (6493), 168–170. doi: 10.1038/371168a0 8072546

[B56] Heffernan B. J. Thomason B. Herring-Palmer A. Hanna P. (2007). *Bacillus anthracis* anthrolysin O and three phospholipases C are functionally redundant in a murine model of inhalation anthrax. FEMS Microbiol. Lett. 271 (1), 98–105. doi: 10.1111/j.1574-6968.2007.00713.x 17419764

[B57] Heffernan B. J. Thomason B. Herring-Palmer A. Shaughnessy L. McDonald R. Fisher N.. (2006). *Bacillus anthracis* phospholipases C facilitate macrophage-associated growth and contribute to virulence in a murine model of inhalation anthrax. Infect. Immun. 74 (7), 3756–3764. doi: 10.1128/IAI.00307-06 16790747 PMC1489738

[B58] Heinz D. W. Essen L. O. Williams R. L. (1998). Structural and mechanistic comparison of prokaryotic and eukaryotic phosphoinositide-specific phospholipases C. J. Mol. Biol. 275 (4), 635–650. doi: 10.1006/jmbi.1997.1490 9466937

[B59] Hemalatha S. G. Drumm B. Sherman P. (1991). Adherence of *Helicobacter pylori* to human gastric epithelial cells in vitro. J. Med. Microbiol. 35 (4), 197–202. doi: 10.1099/00222615-35-4-197 1941988

[B60] Heytens E. Parrington J. Coward K. Young C. Lambrecht S. Yoon S. Y.. (2009). Reduced amounts and abnormal forms of phospholipase C ζ (PLCζ) in spermatozoa from infertile men. Hum. Reprod. 24 (10), 2417–2428. doi: 10.1093/humrep/dep207 19584136

[B61] Hiller M. Lang C. Michel W. Flieger A. (2018). Secreted phospholipases of the lung pathogen *Legionella pneumophila* . Int. J. Med. Microbiol. 308 (1), 168–175. doi: 10.1016/j.ijmm.2017.10.002 29108710

[B62] Hwang J. I. Oh Y. S. Shin K. J. Kim H. Ryu S. H. Suh P. G. (2005). Molecular cloning and characterization of a novel phospholipase C, PLC-η. Biochem. J. 389 (Pt 1), 181–186. doi: 10.1042/BJ20041677 15702972 PMC1184550

[B63] Ichinohe M. Nakamura Y. Sai K. Nakahara M. Yamaguchi H. Fukami K. (2007). Lack of phospholipase C-δ1 induces skin inflammation. Biochem. Biophys. Res. Commun. 356 (4), 912–918. doi: 10.1016/j.bbrc.2007.03.082 17397799

[B64] Iivanainen E. Martikainen P. J. Väänänen P. Katila M. L. (1999). Environmental factors affecting the occurrence of mycobacteria in brook sediments. J. Appl. Microbiol. 86 (4), 673–681. doi: 10.1046/j.1365-2672.1999.00711.x 10212411

[B65] Irino Y. Cho H. Nakamura Y. Nakahara M. Furutani M. Suh P. G.. (2004). Phospholipase C delta-type consists of three isozymes: bovine PLCδ2 is a homologue of human/mouse PLCδ4. Biochem. Biophys. Res. Commun. 320 (2), 537–543. doi: 10.1016/j.bbrc.2004.05.206 15219862

[B66] Jackson A. A. Gross M. J. Daniels E. F. Hampton T. H. Hammond J. H. Vallet-Gely I.. (2013). Anr and its activation by PlcH activity in *Pseudomonas aeruginosa* host colonization and virulence. J. Bacteriol. 195 (13), 3093–3104. doi: 10.1128/JB.02169-12 23667230 PMC3697539

[B67] Jezyk M. R. Snyder J. T. Gershberg S. Worthylake D. K. Harden T. K. Sondek J. (2006). Crystal structure of Rac1 bound to its effector phospholipase C-β2. Nat. Struct. Mol. Biol. 13 (12), 1135–1140. doi: 10.1038/nsmb1175 17115053

[B68] Johansen K. A. Gill R. E. Vasil M. L. (1996). Biochemical and molecular analysis of phospholipase C and phospholipase D activity in mycobacteria. Infect. Immun. 64 (8), 3259–3266. doi: 10.1128/iai.64.8.3259-3266.1996 8757862 PMC174216

[B69] Jones K. T. Matsuda M. Parrington J. Katan M. Swann K. (2000). Different Ca2+-releasing abilities of sperm extracts compared with tissue extracts and phospholipase C isoforms in sea urchin egg homogenate and mouse eggs. Biochem. J. 346 Pt 3 (Pt 3), 743–749. doi: 10.1042/bj3460743 10698702 PMC1220908

[B70] Jones S. Preiter K. Portnoy D. A. (1996). Conversion of an extracellular cytolysin into a phagosome-specific lysin which supports the growth of an intracellular pathogen. Mol. Microbiol. 21 (6), 1219–1225. doi: 10.1046/j.1365-2958.1996.00074.x 8898390

[B71] Kadamur G. Ross E. M. (2013). Mammalian phospholipase C. Annu. Rev. Physiol. 75, 127–154. doi: 10.1146/annurev-physiol-030212-183750 23140367

[B72] Kelley G. G. Reks S. E. Ondrako J. M. Smrcka A. V. (2001). Phospholipase C(ϵ): a novel ras effector. EMBO J. 20 (4), 743–754. doi: 10.1093/emboj/20.4.743 11179219 PMC145421

[B73] Kim E. K. Choi E. J. (2010). Pathological roles of MAPK signaling pathways in human diseases. Biochim. Biophys. Acta 1802 (4), 396–405. doi: 10.1016/j.bbadis.2009.12.009 20079433

[B74] Kim H. K. Kim J. W. Zilberstein A. Margolis B. Kim J. G. Schlessinger J.. (1991). PDGF stimulation of inositol phospholipid hydrolysis requires PLC-γ 1 phosphorylation on tyrosine residues 783 and 1254. Cell 65 (3), 435–441. doi: 10.1016/0092-8674(91)90461-7 1708307

[B75] Kim Y. H. Park T. J. Lee Y. H. Baek K. J. Suh P. G. Ryu S. H.. (1999). Phospholipase C-δ1 is activated by capacitative calcium entry that follows phospholipase C-β activation upon bradykinin stimulation. J. Biol. Chem. 274 (37), 26127–26134. doi: 10.1074/jbc.274.37.26127 10473563

[B76] Kiu R. Hall L. J. (2018). An update on the human and animal enteric pathogen *Clostridium perfringens* . Emerg. Microbes Infect. 7 (1), 141. doi: 10.1038/s41426-018-0144-8 30082713 PMC6079034

[B77] Klein R. R. Bourdon D. M. Costales C. L. Wagner C. D. White W. L. Williams J. D.. (2011). Direct activation of human phospholipase C by its well known inhibitor u73122. J. Biol. Chem. 286 (14), 12407–12416. doi: 10.1074/jbc.M110.191783 21266572 PMC3069444

[B78] Klockgether J. Tümmler B. (2017). Recent advances in understanding *Pseudomonas aeruginosa* as a pathogen. F1000Res 6, 1261. doi: 10.12688/f1000research.10506.1 28794863 PMC5538032

[B79] Kojima M. Hosoda H. Date Y. Nakazato M. Matsuo H. Kangawa K. (1999). Ghrelin is a growth-hormone-releasing acylated peptide from stomach. Nature 402 (6762), 656–660. doi: 10.1038/45230 10604470

[B80] Kong L. Ge B. X. (2008). MyD88-independent activation of a novel actin-Cdc42/Rac pathway is required for toll-like receptor-stimulated phagocytosis. Cell Res. 18 (7), 745–755. doi: 10.1038/cr.2008.65 18542102

[B81] König B. Vasil M. L. König W. (1997). Role of hemolytic and nonhemolytic phospholipase C from *Pseudomonas aeruginosa* for inflammatory mediator release from human granulocytes. Int Arch Allergy Immunol 112 (2), 115–124. doi: 10.1159/000237441 9030090

[B82] Kumari B. Kaur J. Kaur J. (2018). Phospholipases in Bacterial Virulence and Pathogenesis. Adv. Biotechnol. Microbiol. 10 (5), 555798. doi: 10.19080/AIBM.2018.10.555798

[B83] Lagercrantz J. Carson E. Phelan C. Grimmond S. Rosén A. Daré E.. (1995). Genomic organization and complete cDNA sequence of the human phosphoinositide-specific phospholipase C beta 3 gene (PLCβ3). Genomics 26 (3), 467–472. doi: 10.1016/0888-7543(95)80164-h 7607669

[B84] Le Chevalier F. Cascioferro A. Frigui W. Pawlik A. Boritsch E. C. Bottai D.. (2015). Revisiting the role of phospholipases C in virulence and the lifecycle of *Mycobacterium tuberculosis* . Sci. Rep. 5, 16918. doi: 10.1038/srep16918 26603639 PMC4658479

[B85] Leidich S. D. Ibrahim A. S. Fu Y. Koul A. Jessup C. Vitullo J.. (1998). Cloning and disruption of caPLB1, a phospholipase B gene involved in the pathogenicity of candida albicans. J. Biol. Chem. 273 (40), 26078–26086. doi: 10.1074/jbc.273.40.26078 9748287

[B86] Lo Vasco V. R. (2011). Role of phosphoinositide-specific phospholipase C η2 in isolated and syndromic mental retardation. Eur. Neurol. 65 (5), 264–269. doi: 10.1159/000327307 21474938

[B87] Mao G. F. Kunapuli S. P. Koneti Rao A. (2000). Evidence for two alternatively spliced forms of phospholipase C-β2 in haematopoietic cells. Br. J. Haematol. 110 (2), 402–408. doi: 10.1046/j.1365-2141.2000.02201.x 10971398

[B88] Margolis B. Zilberstein A. Franks C. Felder S. Kremer S. Ullrich A.. (1990). Effect of phospholipase C-γ overexpression on PDGF-induced second messengers and mitogenesis. Science 248 (4955), 607–610. doi: 10.1126/science.2333512 2333512

[B89] Marlink K. L. Bacon K. D. Sheppard B. C. Ashktorab H. Smoot D. T. Cover T. L.. (2003). Effects of *Helicobacter pylori* on intracellular Ca2+ signaling in normal human gastric mucous epithelial cells. Am. J. Physiol. Gastrointest Liver Physiol. 285 (1), G163–G176. doi: 10.1152/ajpgi.00257.2002 12606301

[B90] Marquis H. Goldfine H. Portnoy D. A. (1997). Proteolytic pathways of activation and degradation of a bacterial phospholipase C during intracellular infection by *Listeria monocytogenes* . J. Cell Biol. 137 (6), 1381–1392. doi: 10.1083/jcb.137.6.1381 9182669 PMC2132530

[B91] Marshall B. J. Warren J. R. (1984). Unidentified curved bacilli in the stomach of patients with gastritis and peptic ulceration. Lancet 1 (8390), 1311–1315. doi: 10.1016/s0140-6736(84)91816-6 6145023

[B92] Marteyn B. West N. P. Browning D. F. Cole J. A. Shaw J. G. Palm F.. (2010). Modulation of *Shigella* virulence in response to available oxygen in vivo. Nature 465 (7296), 355–358. doi: 10.1038/nature08970 20436458 PMC3750455

[B93] Mattoo S. Yuk M. H. Huang L. L. Miller J. F. (2004). Regulation of type III secretion in *Bordetella* . Mol. Microbiol. 52 (4), 1201–1214. doi: 10.1111/j.1365-2958.2004.04053.x 15130135

[B94] McGregor J. A. Lawellin D. Franco-Buff A. Todd J. K. (1991). Phospholipase C activity in microorganisms associated with reproductive tract infection. Am. J. Obstet Gynecol. 164 (2), 682–686. doi: 10.1016/s0002-9378(11)80046-3 1992722

[B95] Meisenhelder J. Suh P. G. Rhee S. G. Hunter T. (1989). Phospholipase C-γ is a substrate for the PDGF and EGF receptor protein-tyrosine kinases in vivo and *in vitro* . Cell 57 (7), 1109–1122. doi: 10.1016/0092-8674(89)90048-2 2472219

[B96] Meldrum E. Kriz R. W. Totty N. Parker P. J. (1991). A second gene product of the inositol-phospholipid-specific phospholipase C δ subclass. Eur. J. Biochem. 196 (1), 159–165. doi: 10.1111/j.1432-1033.1991.tb15799.x 1848183

[B97] Mengaud J. Dramsi S. Gouin E. Vazquez-Boland J. A. Milon G. Cossart P. (1991). Pleiotropic control of *Listeria monocytogenes* virulence factors by a gene that is autoregulated. Mol. Microbiol. 5 (9), 2273–2283. doi: 10.1111/j.1365-2958.1991.tb02158.x 1662763

[B98] Mengaud J. Vicente M. F. Cossart P. (1989). Transcriptional mapping and nucleotide sequence of the *Listeria monocytogenes* hlyA region reveal structural features that may be involved in regulation. Infect. Immun. 57 (12), 3695–3701. doi: 10.1128/iai.57.12.3695-3701.1989 2509367 PMC259892

[B99] Meyers D. J. Berk R. S. (1990). Characterization of phospholipase C from *Pseudomonas aeruginosa* as a potent inflammatory agent. Infect. Immun. 58 (3), 659–666. doi: 10.1128/iai.58.3.659-666.1990 2106492 PMC258516

[B100] Montes L. R. Ibarguren M. Goñi F. M. Stonehouse M. Vasil M. L. Alonso A. (2007). Leakage-free membrane fusion induced by the hydrolytic activity of PlcHR(2), a novel phospholipase C/sphingomyelinase from *Pseudomonas aeruginosa* . Biochim. Biophys. Acta 1768 (10), 2365–2372. doi: 10.1016/j.bbamem.2007.04.024 17560896

[B101] Monturiol-Gross L. Flores-Díaz M. Pineda-Padilla M. J. Castro-Castro A. C. Alape-Giron A. (2014). *Clostridium perfringens* phospholipase C induced ROS production and cytotoxicity require PKC, MEK1 and NFκB activation. PloS One 9 (1), e86475. doi: 10.1371/journal.pone.0086475 24466113 PMC3900566

[B102] Moreau H. Pieroni G. Jolivet-Reynaud C. Alouf J. E. Verger R. (1988). A new kinetic approach for studying phospholipase C (*Clostridium perfringens* alpha toxin) activity on phospholipid monolayers. Biochemistry 27 (7), 2319–2323. doi: 10.1021/bi00407a012 2898259

[B103] Nagano N. Orengo C. A. Thornton J. M. (2002). One fold with many functions: the evolutionary relationships between TIM barrel families based on their sequences, structures and functions. J. Mol. Biol. 321 (5), 741–765. doi: 10.1016/s0022-2836(02)00649-6 12206759

[B104] Nakahara M. Shimozawa M. Nakamura Y. Irino Y. Morita M. Kudo Y.. (2005). A novel Phospholipase C, PLC(η)2, is a neuron-specific isozyme. J. Biol. Chem. 280 (32), 29128–29134. doi: 10.1074/jbc.M503817200 15899900

[B105] Nakamura Y. Ichinohe M. Hirata M. Matsuura H. Fujiwara T. Igarashi T.. (2008). Phospholipase C-δ1 is an essential molecule downstream of *Foxn1*, the gene responsible for the nude mutation, in normal hair development. FASEB J. 22 (3), 841–849. doi: 10.1096/fj.07-9239com 17938256

[B106] Nakshabendi I. M. Zhang Q. B. Mokhashi M. Gemmell C. G. Lee F. D. Russell R. I. (1996). Effect of omeprazole therapy on the survival of *Helicobacter pylori*, urease activity, and antral gastric histology in patients with duodenal ulcer. Helicobacter 1 (3), 155–158. doi: 10.1111/j.1523-5378.1996.tb00030.x 9398897

[B107] Naylor C. E. Eaton J. T. Howells A. Justin N. Moss D. S. Titball R. W.. (1998). Structure of the key toxin in gas gangrene. Nat. Struct. Biol. 5 (8), 738–746. doi: 10.1038/1447 9699639

[B108] Naylor C. E. Jepson M. Crane D. T. Titball R. W. Miller J. Basak A. K.. (1999). Characterisation of the calcium-binding c-terminal domain of *Clostridium perfringens* alpha-toxin. J. Mol. Biol. 294 (3), 757–770. doi: 10.1006/jmbi.1999.3279 10610794

[B109] Ochsner U. A. Snyder A. Vasil A. I. Vasil M. L. (2002). Effects of the twin-arginine translocase on secretion of virulence factors, stress response, and pathogenesis. Proc. Natl. Acad. Sci. U. S. A. 99 (12), 8312–8317. doi: 10.1073/pnas.082238299 12034867 PMC123064

[B110] Oda M. Ikari S. Matsuno T. Morimune Y. Nagahama M. Sakurai J. (2006). Signal transduction mechanism involved in *Clostridium perfringens* alpha-toxin-induced superoxide anion generation in rabbit neutrophils. Infect. Immun. 74 (5), 2876–2886. doi: 10.1128/IAI.74.5.2876-2886.2006 16622226 PMC1459708

[B111] Oda M. Shiihara R. Ohmae Y. Kabura M. Takagishi T. Kobayashi K.. (2012). *Clostridium perfringens* alpha-toxin induces the release of IL-8 through a dual pathway *via* TrkA in A549 cells. Biochim. Biophys. Acta 1822 (10), 1581–1589. doi: 10.1016/j.bbadis.2012.06.007 22721959

[B112] Osawa H. Nakazato M. Date Y. Kita H. Ohnishi H. Ueno H.. (2005). Impaired production of gastric ghrelin in chronic gastritis associated with *Helicobacter pylori* . J. Clin. Endocrinol. Metab. 90 (1), 10–16. doi: 10.1210/jc.2004-1330 15483107

[B113] Ostroff R. M. Vasil M. L. (1987). Identification of a new phospholipase C activity by analysis of an insertional mutation in the hemolytic phospholipase C structural gene of *Pseudomonas aeruginosa* . J. Bacteriol. 169 (10), 4597–4601. doi: 10.1128/jb.169.10.4597-4601.1987 2820937 PMC213827

[B114] Otterhag L. Sommarin M. Pical C. (2001). N-terminal EF-hand-like domain is required for phosphoinositide-specific phospholipase C activity in *Arabidopsis thaliana* . FEBS Lett. 497 (2-3), 165–170. doi: 10.1016/s0014-5793(01)02453-x 11377433

[B115] Park E. H. Kim J. M. Kim K. M. Kang D. Cho Y. A. Choi J. Y.. (2014). *Helicobacter pylori* γ-glutamyl transpeptidase-induced Ca(2+) release *via* PLC-IP3 receptors in AGS cells. Can. J. Microbiol. 60 (12), 865–868. doi: 10.1139/cjm-2014-0464 25409842

[B116] Paroha R. Chaurasiya S. K. Chourasia R. (2019). Phospholipase C-γ2 promotes intracellular survival of mycobacteria. J. Cell Biochem. 120 (4), 5062–5071. doi: 10.1002/jcb.27783 30317660

[B117] Paroha R. Chourasia R. Rai R. Kumar A. Vyas A. K. Chaurasiya S. K.. (2020). Host phospholipase C-γ1 impairs phagocytosis and killing of mycobacteria by J774A.1 murine macrophages. Microbiol. Immunol. 64 (10), 694–702. doi: 10.1111/1348-0421.12839 32816349

[B118] Pelech S. L. Vance D. E. (1989). Signal transduction via phosphatidylcholine cycles. Trends in Biochemical Sciences 14 (1), 28–30. doi: 10.1016/0968-0004(89)90086-8

[B119] Perskvist N. Zheng L. Stendahl O. (2000). Activation of human neutrophils by *Mycobacterium tuberculosis* H37Ra Involves Phospholipase Cγ2, Shc Adapter Protein, and p38 mitogen-activated protein kinase. J. Immunol. 164 (2), 959–965. doi: 10.4049/jimmunol.164.2.959 10623845

[B120] Piotrowski J. Piotrowski E. Skrodzka D. Slomiany A. Slomiany B. L. (1997). Induction of acute gastritis and epithelial apoptosis by *Helicobacter pylori* lipopolysaccharide. Scand. J. Gastroenterol. 32 (3), 203–211. doi: 10.3109/00365529709000195 9085455

[B121] Portnoy D. A. Chakraborty T. Goebel W. Cossart P. (1992). Molecular determinants of *Listeria monocytogenes* pathogenesis. Infect. Immun. 60 (4), 1263–1267. doi: 10.1128/iai.60.4.1263-1267.1992 1312514 PMC256991

[B122] Poussin M. A. Leitges M. Goldfine H. (2009). The ability of *Listeria monocytogenes* PI-PLC to facilitate escape from the macrophage phagosome is dependent on host PKCβ. Microb. Pathog. 46 (1), 1–5. doi: 10.1016/j.micpath.2008.09.008 18996181 PMC2655198

[B123] Raynaud C. Guilhot C. Rauzier J. Bordat Y. Pelicic V. Manganelli R.. (2002). Phospholipases C are involved in the virulence of *Mycobacterium tuberculosis* . Mol. Microbiol. 45 (1), 203–217. doi: 10.1046/j.1365-2958.2002.03009.x 12100560

[B124] Rhee S. G. Choi K. D. (1992). Regulation of inositol phospholipid-specific phospholipase C isozymes. J. Biol. Chem. 267 (18), 12393–12396. doi: 10.1016/S0021-9258(18)42284-3 1319994

[B125] Ricci V. Giannouli M. Romano M. Zarrilli R. (2014). *Helicobacter pylori* gamma-glutamyl transpeptidase and its pathogenic role. World J. Gastroenterol. 20 (3), 630–638. doi: 10.3748/wjg.v20.i3.630 24574736 PMC3921472

[B126] Rieder G. Hofmann J. A. Hatz R. A. Stolte M. Enders G. A. (2003). Up-regulation of inducible nitric oxide synthase in *Helicobacter pylori*-associated gastritis may represent an increased risk factor to develop gastric carcinoma of the intestinal type. Int. J. Med. Microbiol. 293 (6), 403–412. doi: 10.1078/1438-4221-00280 14760971

[B127] Roche S. M. Grépinet O. Corde Y. Teixeira A. P. Kerouanton A. Témoin S.. (2009). A *Listeria monocytogenes* strain is still virulent despite nonfunctional major virulence genes. J. Infect. Dis. 200 (12), 1944–1948. doi: 10.1086/648402 19911993

[B128] Rock K. L. Gramm C. Rothstein L. Clark K. Stein R. Dick L.. (1994). Inhibitors of the proteasome block the degradation of most cell proteins and the generation of peptides presented on MHC class I molecules. Cell, 78 (5), 761–771. doi: 10.1016/S0092-8674(94)90462-6 8087844

[B129] Ryu S. H. Suh P. G. Cho K. S. Lee K. Y. Rhee S. G. (1987). Bovine brain cytosol contains three immunologically distinct forms of inositolphospholipid-specific phospholipase C. Proc. Natl. Acad. Sci. U. S. A. 84 (19), 6649–6653. doi: 10.1073/pnas.84.19.6649 3477795 PMC299140

[B130] Sato H. Taketomi Y. Murakami M. (2016). Metabolic regulation by secreted phospholipase A_2_ . Inflamm. Regen. 36, 7. doi: 10.1186/s41232-016-0012-7 29259680 PMC5725825

[B131] Saunders C. M. Larman M. G. Parrington J. Cox L. J. Royse J. Blayney L. M.. (2002). PLCζ: a sperm-specific trigger of Ca^2+^ oscillations in eggs and embryo development. Development 129 (15), 3533–3544. doi: 10.1242/dev.129.15.3533 12117804

[B132] Scandella C. J. Kornberg A. (1971). A membrane-bound phospholipase A1 purified from *Escherichia coli* . Biochem. 10 (24), 4447–4456. doi: 10.1021/bi00800a015 4946924

[B133] Schlech W. F Acheson D. (2000). Foodborne Listeriosis. Clin Infect Dis 31(3), 770–775. doi: 10.1086/314008 11017828

[B134] Sekiya F. (2013). Phospholipase C. In Encyclopedia of Biological Chemistry. Elsevier, 467–471. doi: 10.1016/B978-0-12-378630-2.00346-7

[B135] Sener B. Hasçelik G. Ozçelik U. Günalp A. Göçmen A. (1999). Neutrophil chemotaxis in acutely infected and clinically stable cystic fibrosis patients. Pediatr. Int. 41 (5), 514–518. doi: 10.1046/j.1442-200x.1999.01116.x 10530064

[B136] Shimohama S. Homma Y. Suenaga T. Fujimoto S. Taniguchi T. Araki W.. (1991). Aberrant accumulation of phospholipase C-δ in Alzheimer brains. Am. J. Pathol. 139 (4), 737–742. 1928298 PMC1886302

[B137] Shortridge V. D. Lazdunski A. Vasil M. L. (1992). Osmoprotectants and phosphate regulate expression of phospholipase C in *Pseudomonas aeruginosa* . Mol. Microbiol. 6 (7), 863–871. doi: 10.1111/j.1365-2958.1992.tb01537.x 1602966

[B138] Slomiany B. L. Slomiany A. (2015a). Role of amplification in phospholipase Cγ2 activation in modulation of gastric mucosal inflammatory responses to *Helicobacter pylori*: effect of ghrelin. Inflammopharmacology 23 (1), 37–45. doi: 10.1007/s10787-014-0220-1 25362585

[B139] Slomiany B. L. Slomiany A. (2015b). Mechanism of Rac1-induced amplification in gastric mucosal phospholipase Cγ2 activation in response to *Helicobacter pylori*: modulatory effect of ghrelin. Inflammopharmacology 23 (2-3), 101–109. doi: 10.1007/s10787-015-0231-6 25796615

[B140] Smith G. A. Marquis H. Jones S. Johnston N. C. Portnoy D. A. Goldfine H. (1995). The two distinct phospholipases C of *Listeria monocytogenes* have overlapping roles in escape from a vacuole and cell-to-cell spread. Infect. Immun. 63 (11), 4231–4237. doi: 10.1128/iai.63.11.4231-4237.1995 7591052 PMC173601

[B141] Snyder A. Vasil A. I. Zajdowicz S. L. Wilson Z. R. Vasil M. L. (2006). Role of the *Pseudomonas aeruginosa* PlcH tat signal peptide in protein secretion, transcription, and cross-species tat secretion system compatibility. J. Bacteriol. 188 (5), 1762–1774. doi: 10.1128/JB.188.5.1762-1774.2006 16484187 PMC1426547

[B142] Song C. Hu C. D. Masago M. Kariyai K. Yamawaki-Kataoka Y. Shibatohge M.. (2001). Regulation of a novel human phospholipase C, PLCepsilon, through membrane targeting by ras. J. Biol. Chem. 276 (4), 2752–2757. doi: 10.1074/jbc.M008324200 11022048

[B143] Songer J. G. (1997). Bacterial phospholipases and their role in virulence. Trends Microbiol. 5 (4), 156–161. doi: 10.1016/S0966-842X(97)01005-6 9141190

[B144] Stahelin R. V. (2016). “Phospholipid catabolism,” in Biochemistry of lipids, lipoproteins and membranes, Sixth Edition, vol. p. (Elsevier Inc), 237–257.

[B145] Stewart A. J. Mukherjee J. Roberts S. J. Lester D. Farquharson C. (2005). Identification of a novel class of mammalian phosphoinositol-specific phospholipase C enzymes. Int. J. Mol. Med. 15 (1), 117–121. doi: 10.3892/ijmm.15.1.117 15583837

[B146] Stonehouse M. J. Cota-Gomez A. Parker S. K. Martin W. E. Hankin J. A. Murphy R. C.. (2002). A novel class of microbial phosphocholine-specific phospholipases C. Mol. Microbiol. 46 (3), 661–676. doi: 10.1046/j.1365-2958.2002.03194.x 12410824

[B147] Suh P. G. Park J. I. Manzoli L. Cocco L. Peak J. C. Katan M.. (2008). Multiple roles of phosphoinositide-specific phospholipase C isozymes. BMB Rep. 41 (6), 415–434. doi: 10.5483/bmbrep.2008.41.6.415 18593525

[B148] Sukumaran S. K. McNamara G. Prasadarao N. V. (2003). *Escherichia coli* K-1 interaction with human brain micro-vascular endothelial cells triggers phospholipase C-γ1 activation downstream of phosphatidylinositol 3-kinase. J. Biol. Chem. 278 (46), 45753–45762. doi: 10.1074/jbc.M307374200 12952950

[B149] Sundararajan S. Muniyan R. (2021). Latent tuberculosis: interaction of virulence factors in *Mycobacterium tuberculosis* . Mol. Biol. Rep. 48 (8), 6181–6196. doi: 10.1007/s11033-021-06611-7 34351540

[B150] Swann K. Saunders C. M. Rogers N. T. Lai F. A. (2006). PLCζ(zeta): a sperm protein that triggers Ca^2+^ oscillations and egg activation in mammals. Semin. Cell Dev. Biol. 17 (2), 264–273. doi: 10.1016/j.semcdb.2006.03.009 16730199

[B151] Tanti J. F. Jager J. (2009). Cellular mechanisms of insulin resistance: role of stress-regulated serine kinases and insulin receptor substrates (IRS) serine phosphorylation. Curr. Opin. Pharmacol. 9 (6), 753–762. doi: 10.1016/j.coph.2009.07.004 19683471

[B152] Tattoli I. Sorbara M. T. Yang C. Tooze S. A. Philpott D. J. Girardin S. E. (2013). *Listeria* phospholipases subvert host autophagic defenses by stalling pre-autophagosomal structures. EMBO J. 32 (23), 3066–3078. doi: 10.1038/emboj.2013.234 24162724 PMC3844955

[B153] Terada L. S. Johansen K. A. Nowbar S. Vasil A. I. Vasil M. L. (1999). *Pseudomonas aeruginosa* hemolytic phospholipase C suppresses neutrophil respiratory burst activity. Infect. Immun. 67 (5), 2371–2376. doi: 10.1128/IAI.67.5.2371-2376.1999 10225897 PMC115980

[B154] Tidyman W. E. Rauen K. A. (2009). The RASopathies: Developmental syndromes of Ras/MAPK pathway dysregulation. Curr. Opin. Genet. Dev. 19 (3), 230–236. doi: 10.1016/j.gde.2009.04.001 19467855 PMC2743116

[B155] Tilney L. G. Portnoy D. A. (1989). Actin filaments and the growth, movement, and spread of the intracellular bacterial parasite, *Listeria monocytogenes* . J. Cell Biol. 109 (4 Pt 1), 1597–1608. doi: 10.1083/jcb.109.4.1597 2507553 PMC2115783

[B156] Titball R. W. (1993). Bacterial phospholipases C. Microbiol. Rev. 57 (2), 347–366. doi: 10.1128/mr.57.2.347-366.1993 8336671 PMC372913

[B157] Titball R. W. (1998). Bacterial phospholipases. Symposium Series (Society for Applied Microbiology) 27, 127S–137S. 9750370

[B158] Vasil M. L. Stonehouse M. J. Vasil A. I. Wadsworth S. J. Goldfine H. Bolcome R. E. 3rd. (2009). A complex extracellular sphingomyelinase of *Pseudomonas aeruginosa* inhibits angiogenesis by selective cytotoxicity to endothelial cells. PloS Pathog. 5 (5), e1000420. doi: 10.1371/journal.ppat.1000420 19424430 PMC2673038

[B159] Vasquez A. M. Mouchlis V. D. Dennis E. A. (2018). Review of four major distinct types of human phospholipase A_2_ . Adv. Biol. Regul. 67, 212–218. doi: 10.1016/j.jbior.2017.10.009 29248300 PMC5807221

[B160] Verkleij A. J. Zwaal R. F. Roelofsen B. Comfurius P. Kastelijn D. van Deenen L. L. (1973). The asymmetric distribution of phospholipids in the human red cell membrane. a combined study using phospholipases and freeze-etch electron microscopy. Biochim. Biophys. Acta 323 (2), 178–193. doi: 10.1016/0005-2736(73)90143-0 4356540

[B161] Vines C. M. (2012). Phospholipase C. In Islam S. ed. Calcium Signaling. Advances in Experimental Medicine and Biology. Dordrecht: Springer Netherlands, 235–254. doi: 10.1007/978-94-007-2888-2_10 22453945

[B162] Wahl M. I. Daniel T. O. Carpenter G. (1988). Antiphosphotyrosine recovery of phospholipase C activity after EGF treatment of a-431 cells. Science 241 (4868), 968–970. doi: 10.1126/science.2457254 2457254

[B163] Waldo G. L. Ricks T. K. Hicks S. N. Cheever M. L. Kawano T. Tsuboi K.. (2010). Kinetic scaffolding mediated by a phospholipase C-β and Gq Signaling Complex. Science 330 (6006), 974–980. doi: 10.1126/science.1193438 20966218 PMC3046049

[B164] Wang C. K. Pan L. Chen J. Zhang M. (2010). Extensions of PDZ domains as important structural and functional elements. Protein Cell. 1 (8), 737–751. doi: 10.1007/s13238-010-0099-6 21203915 PMC4875201

[B165] Wargo M. J. Gross M. J. Rajamani S. Allard J. L. Lundblad L. K. Allen G. B.. (2011). Hemolytic phospholipase C inhibition protects lung function during *Pseudomonas aeruginosa* infection. Am. J. Respir. Crit. Care Med. 184 (3), 345–354. doi: 10.1164/rccm.201103-0374OC 21562128 PMC3175536

[B166] Wargo M. J. Ho T. C. Gross M. J. Whittaker L. A. Hogan D. A. (2009). GbdR regulates *Pseudomonas aeruginosa* plcH and pchP transcription in response to choline catabolites. Infect. Immun. 77 (3), 1103–1111. doi: 10.1128/IAI.01008-08 19103776 PMC2643652

[B167] Warren J. R. Marshall B. (1983). Unidentified curved bacilli on gastric epithelium in active chronic gastritis. Lancet 1 (8336), 1273–1275. doi: 10.1016/S0140-6736(83)92719-8 6134060

[B168] Waseem T. Duxbury M. Ito H. Ashley S. W. Robinson M. K. (2008). Exogenous ghrelin modulates release of pro-inflammatory and anti-inflammatory cytokines in LPS-stimulated macrophages through distinct signaling pathways. Surgery 143 (3), 334–342. doi: 10.1016/j.surg.2007.09.039 18291254 PMC2278045

[B169] Wayne L. G. Sohaskey C. D. (2001). Nonreplicating persistence of *Mycobacterium tuberculosis* . Annu. Rev. Microbiol. 55, 139–163. doi: 10.1146/annurev.micro.55.1.139 11544352

[B170] Weingart C. L. Hooke A. M. (1999a). A nonhemolytic phospholipase C from *Burkholderia cepacia* . Curr. Microbiol. 38 (4), 233–238. doi: 10.1007/pl00006793 10069860

[B171] Weingart C. L. Hooke A. M. (1999b). Regulation of expression of the nonhemolytic phospholipase C of *Burkholderia cepacia* . Curr. Microbiol. 39 (6), 336–0341. doi: 10.1007/s002849900468 10525838

[B172] Wetherall B. L. Johnson A. M. (1989). Haemolytic activity of *Campylobacter pylori* . Eur. J. Clin. Microbiol. Infect. Dis. 8 (8), 706–710. doi: 10.1007/BF01963756 2506037

[B173] White M. J. Boyd J. M. Horswill A. R. Nauseef W. M. (2014). Phosphatidylinositol-specific phospholipase C contributes to survival of *Staphylococcus aureus* USA300 in human blood and neutrophils. Infect. Immun. 82 (4), 1559–1571. doi: 10.1128/IAI.01168-13 24452683 PMC3993399

[B174] Williams R. L. (1999). Mammalian phosphoinositide-specific phospholipase C. Biochimica et Biophysica Acta (BBA) - Molecular and Cell Biology of Lipids 1441 (2-3), 255–267. doi: 10.1016/S1388-1981(99)00150-X 10570253

[B175] Williams T. J. (1979). Prostaglandin E2, prostaglandin I2 and the vascular changes of inflammation. Br. J. Pharmacol. 65 (3), 517–524. doi: 10.1111/j.1476-5381.1979.tb07860.x 371730 PMC1668629

[B176] Wilton D.C. (2008). Phospholipases. In Vance D. E. Vance J. E. eds. Biochemistry of Lipids, Lipoproteins and Membranes. Elsevier.

[B177] Worlitzsch D. Tarran R. Ulrich M. Schwab U. Cekici A. Meyer K. C.. (2002). Effects of reduced mucus oxygen concentration in airway pseudomonas infections of cystic fibrosis patients. J. Clin. Invest. 109 (3), 317–325. doi: 10.1172/JCI13870 11827991 PMC150856

[B178] Wortzel I. Seger R. (2011). The ERK cascade: Distinct functions within various subcellular organelles. Genes Cancer 2 (3), 195–209. doi: 10.1177/1947601911407328 21779493 PMC3128630

[B179] Wu W. Jin Y. Bai F. Jin S. (2015). “ Pseudomonas aeruginosa ,” in Molecular medical microbiology, 2nd ed. (Elsevier Ltd), 753–767. doi: 10.1016/B978-0-12-397169-2.00041-X

[B180] Yadav M. Clark L. Schorey J. S. (2006). Macrophage's proinflammatory response to a mycobacterial infection is dependent on sphingosine kinase-mediated activation of phosphatidylinositol phospholipase C, protein kinase C, ERK1/2, and phosphatidylinositol 3-kinase. J. Immunol. 176 (9), 5494–5503. doi: 10.4049/jimmunol.176.9.5494 16622018

[B181] Yagisawa H. Tanase H. Nojima H. (1991). Phospholipase C-δ gene of the spontaneously hypertensive rat harbors point mutations causing amino acid substitutions in a catalytic domain. J. Hypertens. 9 (11), 997–1004. doi: 10.1097/00004872-199111000-00004 1684614

[B182] Yoon S. S. Hennigan R. F. Hilliard G. M. Ochsner U. A. Parvatiyar K. Kamani M. C.. (2002). *Pseudomonas aeruginosa* anaerobic respiration in biofilms: relationships to cystic fibrosis pathogenesis. Dev. Cell. 3 (4), 593–603. doi: 10.1016/s1534-5807(02)00295-2 12408810

[B183] Yoon S. Y. Jellerette T. Salicioni A. M. Lee H. C. Yoo M. S. Coward K.. (2008). Human sperm devoid of PLC, zeta 1 fail to induce Ca^2+^ release and are unable to initiate the first step of embryo development. J. Clin. Invest. 118 (11), 3671–3681. doi: 10.1172/JCI36942 18924610 PMC2567839

[B184] Zhou Y. Wing M. R. Sondek J. Harden T. K. (2005). Molecular cloning and characterization of PLC-η2. Biochem. J. 391 (Pt 3), 667–676. doi: 10.1042/BJ20050839 16107206 PMC1276968

[B185] Zhou X. Ye Y. Sun Y. Li X. Wang W. Privratsky B.. (2015). Transient receptor potential channel 1 deficiency impairs host defense and proinflammatory responses to bacterial infection by regulating protein kinase Cα signaling. Mol. Cell Biol. 35 (16), 2729–2739. doi: 10.1128/MCB.00256-15 26031335 PMC4508326

